# Similarity of therapeutic networks induced by a multi-component herbal remedy, Ukgansan, in neurovascular unit cells

**DOI:** 10.1038/s41598-020-59537-8

**Published:** 2020-02-14

**Authors:** Bu-Yeo Kim, Hye-Sun Lim, Yu Jin Kim, Eunjin Sohn, Yun Hee Kim, Imhoi Koo, Soo-Jin Jeong

**Affiliations:** 1 Herbal Medicine Research Division, Korea Institute of Oriental Medicine, 1672 Yuseong-daero, Yuseong-gu Daejeon, 34054 Republic of Korea; 2 College of Pharmacy, Chungnam National University, 99 Daehak-ro, Yuseong-gu Daejeon, 34134 Republic of Korea; 3 Huck Institutes of Life Sciences, Pennsylvania State University, University Park, PA 16802 United States of America

**Keywords:** Networks and systems biology, Gene regulatory networks, High-throughput screening

## Abstract

The neurovascular unit, which includes neurons, glial cells, and vascular cells, plays crucial roles in the onset and progression of Alzheimer’s disease (AD). Therefore, effective drugs against AD should be able to target the multi-cellular neurovascular unit and the therapeutic relationships among neurovascular cells should be defined. Here, we examined the therapeutic effects of Ukgansan (UGS), an herbal remedy with multi-targeting capabilities, using *in vitro* neurovascular unit models and an *in vivo* model of AD. In addition, we compared the therapeutic networks induced by UGS and its components in different neurovascular cell types. We found that UGS and its components protected neurovascular cells against diverse damaging agents and improved the behavioral patterns of AD model mice. A comparison of UGS- or its components-induced therapeutic networks, constructed from high-throughput data on gene expression, pathway activity, and protein phosphorylation, revealed similarities among neurovascular cell types, especially between BV-2 microglia and HBVP (human brain vascular pericytes). These findings, together with the functional connections between neurovascular cells, can explain the therapeutic effects of UGS. Furthermore, they suggest underlying similarities in the therapeutic mechanisms in different neurovascular cell types.

## Introduction

Alzheimer’s disease (AD), characterized by cognitive decline and memory impairment, is a progressive and chronic neurological disorder occurring primarily in the elderly, aged 65 years or older^1^. The main pathological characteristics of AD include the extracellular deposition of the β-amyloid (Aβ) peptides in the brain^2,3^. But recent failures of clinical trials of new drugs targeting Aβ have questioned the precise biological role and importance of Aβ in the pathological process of AD^4,5^. On the other hand, abnormalities of the immune system, mitochondrial dysfunctions, and reactive oxygen species (ROS) generated therefrom in the brain have been actively investigated as other major causes of AD^6,7^, indicating the presence of diverse causal factors in its development and progression.

Moreover, recent reports have suggested that the onset and progression of AD should be considered in terms of the neurovascular unit. In this multicellular complex^8,9^, neurons, glial cells, and vascular cells communicate with each other to tightly regulate the functional homeostasis of the brain^10^. For example, activation of neurovascular pathogenic pathways has been shown to impair synaptic and neuronal functions in parallel with Aβ accumulation and intraneuronal tangle formation, leading to neuronal loss and AD^11^. Moreover, the neurovascular unit regulates cerebral blood flow^12,13^, thus playing a critical role in the onset and progression of AD.

Diverse factors have been reported to stimulate the neurovascular unit, resulting in pathological conditions. For example, ROS play a crucial role in neuronal damage induced by Aβ1^14^. In this process, activation of microglia also occurs through signal pathways involving toll-like receptor 4, interleukins (ILs), and chemokines, most of which are also commonly stimulated by lipopolysaccharide (LPS)^15^. On the other hand, cerebral hypoperfusion/hypoxia condition activates vascular cells and stimulates the angiogenic process, resulting in the dysregulation of the neurovascular system and finally leading to degenerative changes in the blood–brain barrier (BBB)^16^. It has also been reported that transforming growth factor (TGF)-β1 regulates brain pericyte inflammation involved in BBB functions^17^. Therefore, during the development of the pathological conditions related to AD, neurovascular systems are exposed to various types of environments. Emerging evidence for the important roles of multicellular integration in AD pathogenesis strongly suggests the necessity for novel drugs capable of targeting multiple cell types.

Ukgansan (UGS), also called Yokukansan or Yi-Gan San, is a traditional oriental prescription composed of 7 medicinal ingredients including *Uncaria sinensis* (Uncariae Ramulus et Uncus)*, Atractylodes japonica* (Atractylodis Rhizoma Alba)*, Poria cocos* (Poria Sclerotium)*, Bupleurum falcatum* (Bupleuri Radix)*, Angelica gigas* (Angelicae Gigantis Radix)*, Cnidium officinale* (Cnidii Rhizoma), and *Glycyrrhiza uralensis* (Glycyrrhizae Radix et Rhizoma)^18^. UGS has been approved by the Ministry of Health, Labour and Welfare of Japan for use against pathological conditions such as insomnia, irritability, and neurosis in children^19^. In addition, UGS has been reported to improve behavioral deficits and protect neuronal cells from degeneration in animal models^20–22^. We also demonstrated that ferulic acid, one of the active compounds of UGS, could play an important role, as an antioxidant, in its therapeutic effects^18^. These previous reports strongly suggest that UGS could exert various therapeutic roles in the brain by targeting diverse cellular components. However, the exact molecular mechanisms are unclear.

Indeed, one of the potential advantages of herbal drugs in disease treatment is the multi-targeting ability and therapeutic complementarity allowed by their diverse herbal components. However, identifying the biological targets and interaction mechanisms of each individual chemical component is challenging, due to the tremendously complex chemical nature of herbal drugs. Nevertheless, synergistic mechanisms in molecular actions between herbal chemicals were suggested as a possible therapeutic mechanism of herbal drugs^23^, based on the concept of complementarity in the combination of chemical components.

Despite such multi-targeting properties and complementarity among herbal components, most herbal medicine research has been focused on the identification of single active components acting on a few biological targets, such as individual genes and proteins physically interacting with the major chemical components of herbal drugs^24–26^. However, identifying only a small number of chemical components and their corresponding biological targets cannot adequately describe the whole therapeutic action of herbal drugs. Rather, we hypothesized that these multi-targeting properties of herbal drugs could be the main factor explaining their therapeutic effectiveness against diverse diseases. In recent years, many herbal medicine studies applied network-based approaches to overcome the lack of information on the targets of the identified herbal constituents^27,28^. We also reported that combining omics and pharmacogenomics network approaches can reveal the therapeutic properties of herbal drugs^29,30^. However, unfortunately, most network-based studies of herbal drugs are based on limited experimental evidence that does not fully reflect the diverse aspects of the disease.

In the present study, we aimed to examine the therapeutic effects of UGS and its components using *in vitro* neurovascular unit models and an *in vivo* model of Aβ-induced AD. We also used high-throughput data on gene expression, pathway activity, and protein phosphorylation to compare the therapeutic networks induced by UGS and its components in different neurovascular cell types. The results described below provide novel information about the therapeutic mechanisms of UGS. We also expect that our approach based on the analysis of therapeutic patterns by multiple drug components could be applied to the assessment of drug efficacy in other complex pathological conditions involving diverse cell types.

## Results

### Composition of UGS

UGS is composed of 7 individual components including *Uncaria sinensis* (C1)*, Atractylodes japonica* (C2)*, Poria cocos* (C3)*, Bupleurum falcatum* (C4)*, Angelica gigas* (C5)*, Cnidium officinale* (C6), and *Glycyrrhiza uralensis* (C7). The composition and content of each UGS herbal component is shown in Table 1. In addition to the 7 individual herbal components of UGS, 3 mixtures composed of 2 herbal components each were also prepared to increase the number of herbal combinations: Mix1 contained C1 and C2, Mix2 contained C3 and C4, and Mix3 contained C5 and C6 (Table 1). The composition of the mixtures was determined based on the biological and pharmacological properties of the 7 individual components (Supplementary Table S1). Specifically, each herbal component (C1–C6) was annotated as having neuroprotective and/or anti-neuroinflammatory effects. We produced only three mixture groups (Mix1, Mix2 and Mix3) as a pilot study before considering all possible combinations.

**Table 1:** Herbal composition of UGS.

Latin name	Symbol	Scientific name	Amount (g)	Origin
Uncariae Ramulus et Uncus	C1	*Uncaria sinensis*	6	China
Atractylodis Rhizoma Alba	C2	*Atractylodes japonica*	8	China
Poria Sclerotium	C3	*Poria cocos*	8	China
Bupleuri Radix	C4	*Bupleurum falcatum*	4	China
Angelicae Gigantis Radix	C5	*Angelica gigas*	6	Korea
Cnidii Rhizoma	C6	*Cnidium officinale*	6	China
Glycyrrhizae Radix et Rhizoma	C7	*Glycyrrhiza uralensis*	3	China
**Mixture symbol**	**Composition**	**Ratio in Mixture (g)**		
Mix1	C1 + C2	3 + 4		
Mix2	C3 + C4	2 + 1		
Mix3	C5 + C6	1 + 1		

### *In vitro* measurement of the anti-damage effects of UGS in neurovascular cell systems

**Figure 1:**
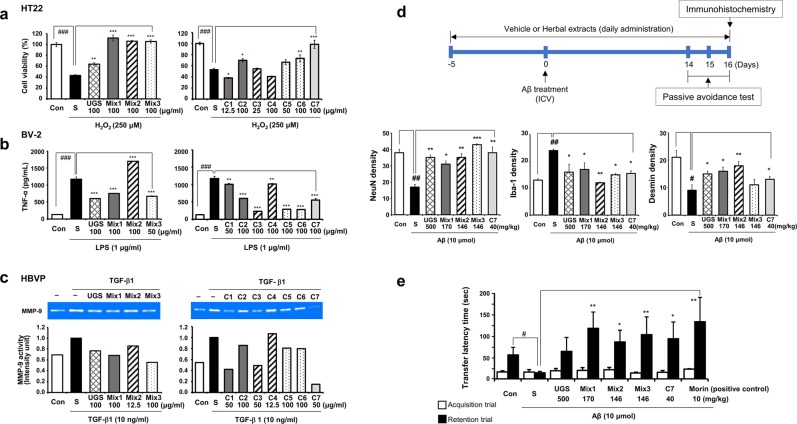
Effects of UGS and its herbal components on the alteration of the neurovascular cell unit and an Aβ-induced mouse model of Alzheimer’s disease. (**a**) HT22 hippocampal cells were treated with H_2_O_2_ (250 μM) and each herbal extract for 6 h. Cytotoxicity was determined by CCK assay. The left panel presents the effects of UGS and the 3 herbal component mixtures (Mix), and the right panel presents the effects of individual herbal components (C1–C7). The white bar (Con) represents untreated control samples and the black bar (S) represents H_2_O_2_-stimulated samples. The patterns of the bar graph reflect the composition of the herbal drugs, as shown in Table 1. For example, the graph pattern of Mix1 matches that of C1 and C2; Mix2 matches C3 and C4; and Mix 3 matches C5 and C6. (**b**) BV-2 microglial cells were pretreated with each herbal extract for 2 h and then stimulated with LPS (1 μg/mL) for an additional 22 h. The supernatants were collected and applied to ELISA for TNF-α. The display format of the graph is the same as that of (a). Data represent the mean ± standard error of 3 independent experiments. ^###^p < 0.01 vs untreated cells; *p < 0.05, **p < 0.01 or ***p < 0.001 vs stimulated cells by H_2_O_2_ or LPS. (**c**) HBVP pericytes were treated with TGF-β1 (10 ng/mL) and each herbal extract for 24 h. MMP-9 enzymatic activity was assessed by gelatin zymography using the culture supernatants of each sample. Gelatinase activity was detected as a distinct clear area on a blue stained gelatin background. The display format of the graph is the same as that of (a). Full-length gels are presented in Supplementary Fig. S1. (**d**) Upper panel shows the scheme of animal experiment. For the immunohistochemistry (IHC) assay, Aβ aggregates (10 μmol per 10% dimethyl sulfoxide in PBS) were injected into the intracerebroventricular (ICV) region of ICR mice in stereotaxic coordinates. Vehicle or each herbal extract was orally administered for 21 days. Expressions of NeuN and desmin in the hippocampus and Iba-1 in the cortex were determined by IHC and then quantified using ImageJ. Detailed images of IHC are shown in Supplementary Fig. S3. Data represent the mean ± standard error of 3 independent experiments. ^###^p < 0.01 vs untreated samples; *p < 0.05, **p < 0.01 or ***p < 0.001 vs Aβ-stimulated samples. (**e**) In the passive avoidance task test, mice were trained in a single step passive avoidance task 14 days after Aβ injection (retention trial). The testing trial was given 2 days later (acquisition trial). The latency was recorded both at the acquisition and retention trials. Morin was administered as a positive control. Data represent the mean ± standard error of 3 independent experiments.

Given the diversity of factors reported to lead the neurovascular unit towards a pathological state^31^, we applied various stimulants to mimic the diverse pathological conditions occurring in the neurovascular system of patients with AD. HT22 hippocampal cells were exposed to H_2_O_2_ to induce neuronal cell death and co-treated with each herbal component. H_2_O_2_ treatment significantly reduced cell viability compared to untreated controls, whereas UGS significantly reversed the effect of H_2_O_2_ on neuronal cell death. Mix1, Mix2, and Mix3 also significantly inhibited H_2_O_2_-induced cell death of HT22 hippocampal cells. Among the 7 single herbal components, C2, C6, and C7 displayed protective effects against neuronal cell death (Fig. 1a).

BV-2 microglia were stimulated with LPS to induce an inflammatory reaction, with or without each herbal component. LPS stimulation significantly increased the amount of inflammatory cytokine tumor necrosis factor (TNF)-α compared to untreated controls. UGS, Mix1, and Mix3 significantly decreased LPS-mediated TNF-α production; in contrast, Mix2 further increased it. All 7 single herbal components reduced LPS-mediated TNF-α production, and the effects of C3, C5, and C6 were the most pronounced (Fig. 1b). In addition to TNF-α, we also measured the level of IL-6, an immunological marker that responds to LPS, in BV-2 microglia. UGS and its herbal components significantly decreased LPS-mediated IL-6 production. Some of the single herbal components (C3–C6) affected IL-6 and TNF-α levels differently, whereas others (UGS, Mix1–3, C1, C2, and C7) had similar effects (Supplementary Fig. S2).

The inflammatory cascade mediates the production by pericytes of matrix metalloproteinase (MMP)^32^, a key mediator of BBB disruption^33^. TGF-β1–induced MMP-9 upregulation has been reported to be important in pericyte–endothelial cell communication^34^. In the present study, the effects of UGS and its herbal components on MMP-9 production were evaluated using a zymographic assay in TGF-β1-damaged human brain vascular pericyte (HBVP) cells. TGF-β1 treatment increased the level of MMP-9 compared to untreated cells. Co-treatment of TGF-β1 with UGS, Mix1, Mix2, and Mix3 slightly reduced the MMP-9 levels induced by TGF-β1. Among the 7 single herbal components, C1, C3, C5, C6 and C7 suppressed the increase of MMP-9 production induced by TGF-β1 (Fig. 1c).

A mouse model of AD was obtained by injecting Aβ aggregates into the intracerebroventricular (ICV) region of the brain^35,36^. Oral administration of herbal components was initiated 5 days before Aβ injection and continued for a total of 21 days to study the preventive as well as the curative effects. Immunohistochemistry (IHC) analysis was performed to determine the effects of UGS and its herbal components on the neurovascular unit of the animal model (Fig. 1d). Hippocampal expression of NeuN, a neuronal marker, and desmin, a pericyte marker^37–39^, was reduced by Aβ injection compared to the control group. By contrast, UGS, Mix1, Mix2, and C7 blocked the effect of Aβ on NeuN and desmin expression (p < 0.05). In addition, Aβ treatment activated cortical expression of Iba-1, a microglia marker, compared to the control group. UGS and all components clearly reversed the Aβ-induced induction of Iba-1 expression (p < 0.05). Detailed images of IHC are shown in Supplementary Fig. S3.

We also evaluated the effects of each herbal component on memory improvement in our Aβ-injected AD mouse model, using the passive avoidance task test. ICV injection of Aβ aggregates significantly shortened the passive avoidance latency in treated animals compared with controls (p < 0.05). By contrast, Mix1, Mix2, Mix3, and C7 treatment significantly increased such latency compared with animals from the Aβ group (p < 0.01 or 0.001, Fig. 1e). Morin was used as a positive control^40^. UGS also improved the behavioral patterns of the AD model mice, but the statistical significance was marginal (p = 0.07).

Since the treatment concentration of each component was determined as the dose showing maximal therapeutic effects without toxicity, the doses of UGS and its herbal components were different in each experimental condition. Therefore, we could not directly compare the efficacies of herbal components within and between cell types. Instead, we focused on the therapeutic patterns of herbal drugs. Supplementary Fig. S4 presents the correlation plots between neurovascular cell types based on the efficacy distribution of herbal drugs. BV-2 microglial cells and HBVP pericytes showed similar therapeutic response patterns (r = 0.30), although without statistical significance (p = 0.28).

### Functional implications of genes in neurovascular cells

Although we confirmed the therapeutic efficacy of UGS and its components using *in vivo* and *in vitro* experiments, the detailed therapeutic mechanisms of the herbal components remained to be clarified. Therefore, we measured the molecular effects of each stimulant and herbal component on the regulation of gene expression in neurovascular cells using quantitative whole mRNA sequencing methods.

**Figure 2:**
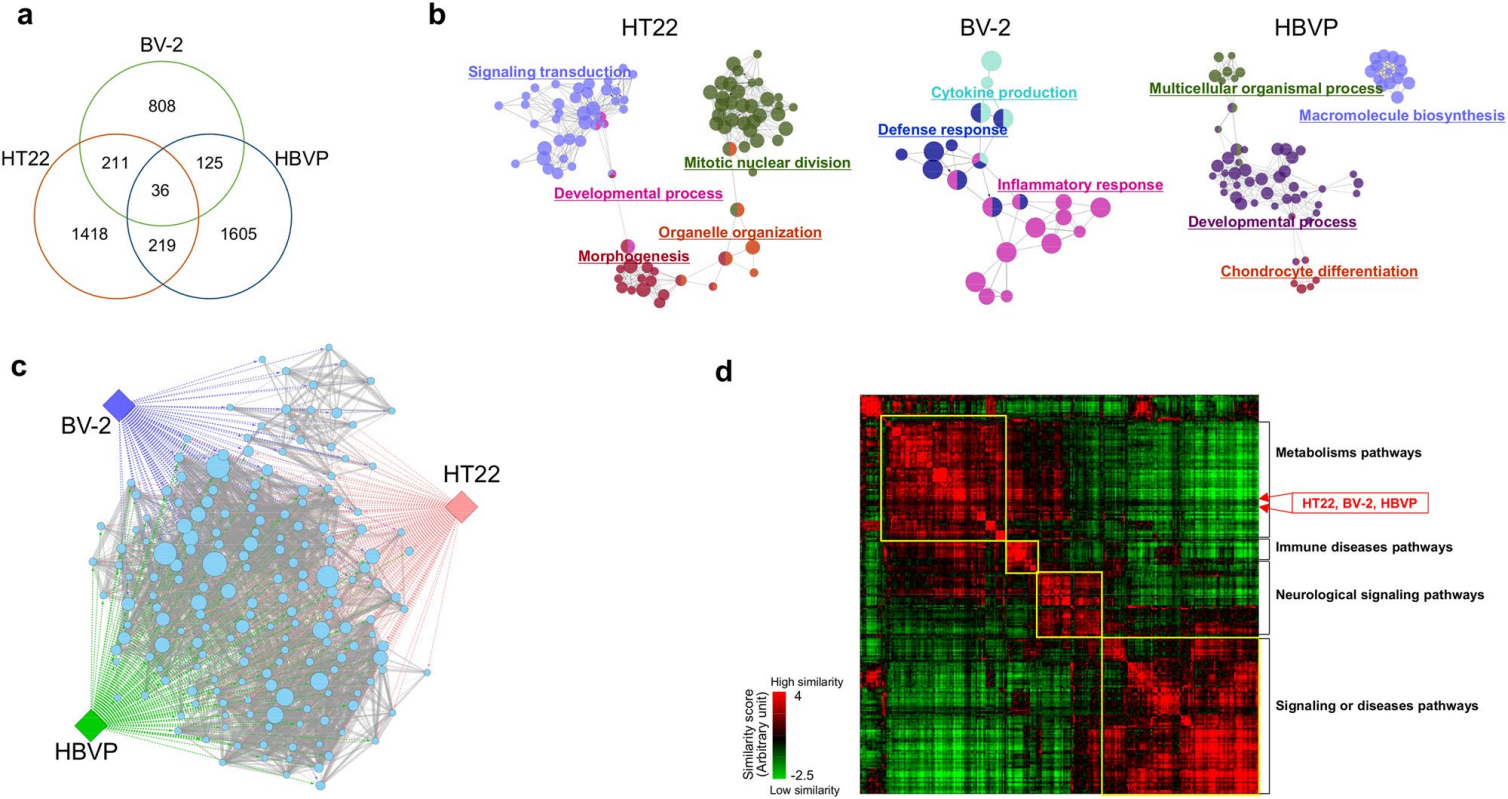
Functional characteristics of genes from stimulated neurovascular cells. HT22 hippocampal cells, BV-2 microglia, and HBVP pericytes were exposed to H_2_O_2_, LPS, and TGF-β1, respectively. Total RNA extracts from cultivated cells and mouse hippocampi were analyzed by RNA-seq technology. Gene expression levels were compared with those of control cells to obtain expression ratios. Genes showing expression ratios above 2 or below 0.5 in each experimental condition were identified as DEGs. (**a**) The distribution of DEGs among neurovascular cell types is shown as a Venn diagram. (**b**) The functional characterization of DEGs from each stimulated neurovascular cells (HT22, BV-2, and HBVP) was assessed using ClueGO. The GO terms enriched in each stimulated neurovascular cell type were clustered (FDR < 0.01) and represented with the same color. Representative GO terms for each cluster are shown. The size of each node indicates the enrichment significance of the GO term. (**c**) GO terms associated with DEGs from each stimulated neurovascular cell type were identified with DAVID and redundancies removed using ReviGO. The resultant GO terms were connected with each other based on common genes (p < 0.001) using the Enrichment Map. The diamond and circle represent neurovascular cell type and GO terms, respectively. Circle node size and edge thickness represent the number of genes included in a GO term and shared by two GO terms, respectively. A detailed image with GO terms is shown in Supplementary Fig. S5. (**d**) DEGs from neurovascular cells were mapped into a PPI network. Based on the shortest path length of the DEGs to each gene set of 281 pathways, the position of neurovascular cell types were determined, indicated with red arrows. Representative biological functions of each cluster of pathways are shown in the right side of the image.

Genes showing expression fold changes greater than 2 or less than 0.5 compared with controls were considered differentially expressed genes (DEGs) in each experimental condition. The distribution of DEGs among neurovascular cells is shown in Fig. 2a. The number of genes changing in response to each stimulant indicated the genes induced differentially in each neurovascular cell type. The biological functions associated with the DEGs from each stimulated neurovascular cell type were represented as a Gene Ontology (GO) network using ClueGO^41^, as shown in Fig. 2b. Based on the differences in commonly responding genes, diverse sets of GO terms were enriched in each type of neurovascular cells, such as mitotic nuclear division in HT22 hippocampal cells, inflammatory response in BV-2 microglia, and developmental regulation in HBVP cells. Then we observed the functional connections among GO terms by applying the Enrichment Map^42^ and ReviGO^43^. Figure 2c shows that each independently enriched GO terms from the different cell types were functionally connected with each other, supporting the biological relevance of the responses of the 3 types of neurovascular cells against diverse stimuli. A detailed image of Fig. 2c is shown in Supplementary Fig. S5. We also observed the same functional consistency between hippocampal tissues from Aβ-administered mice and neurovascular cells, as shown in Supplementary Fig. S6. Figure 2d shows that DEGs from each neurovascular cell type clustered together into a large group of pathways, mainly composed of metabolism-related pathways, in a protein-protein interaction (PPI)-based functional network analysis. This result also indicates the presence of commonly implicated functions in response to different stimuli in neurovascular cells. However, at the level of individual genes, the number of commonly responding genes between neurovascular cell types was relatively small: only 36 genes (at most approximately 3%) were shared among cell types (Fig. 2a), indicating that approaches based on individual genes should be applied with caution in studies investigating different sources of biological samples.

### Changes in gene expression induced by UGS and its herbal components

**Figure 3:**
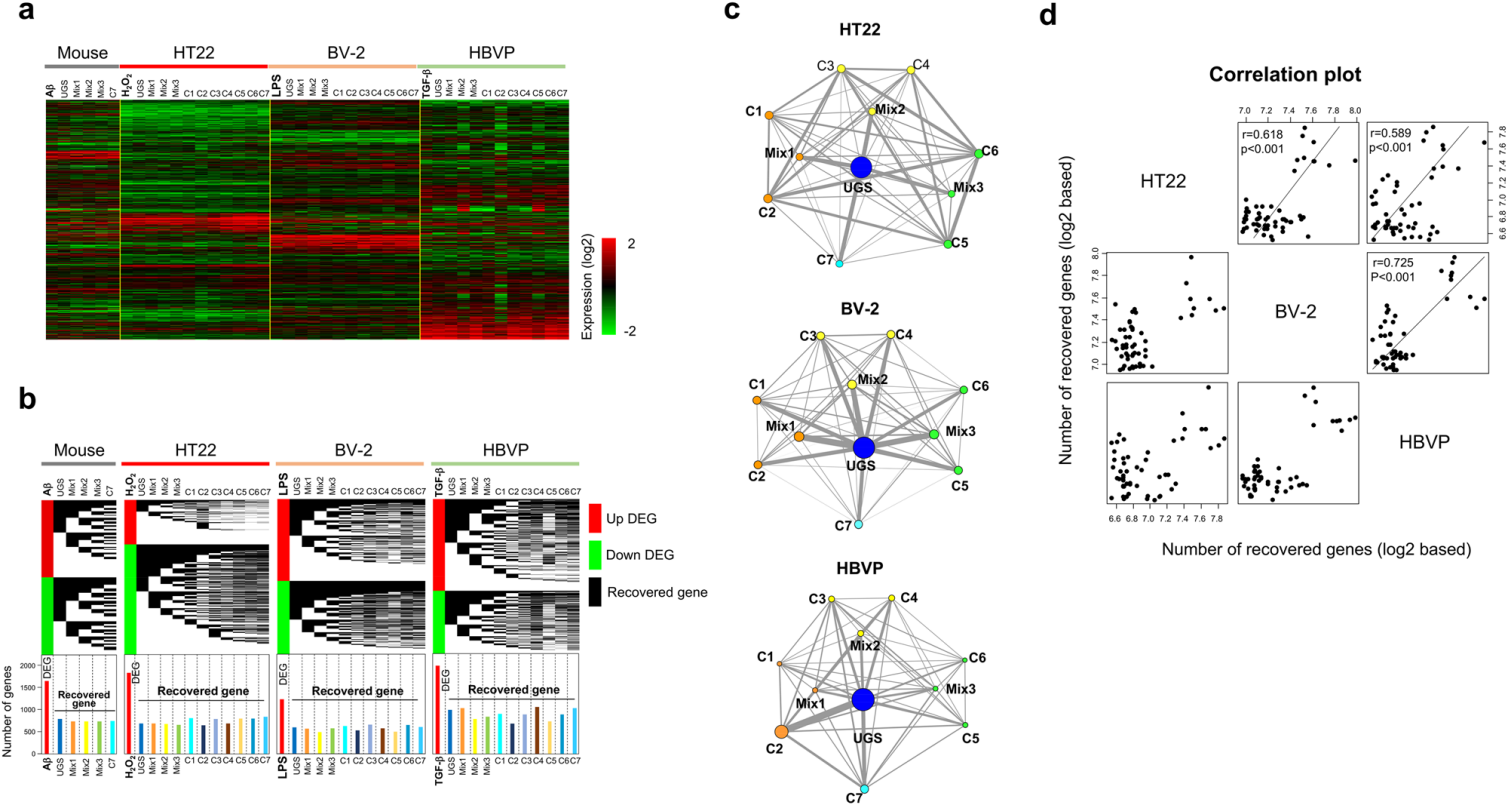
Therapeutic similarities between neurovascular cells based on gene expression. Gene expression ratios were measured from HT22 hippocampal cells, BV-2 microglia, and HBVP pericytes exposed to H_2_O_2_, LPS, and TGF-β1, respectively, by RNA-seq technology. To obtain hippocampal tissue, mice were injected with Aβ into the ICV region. (**a**) An expression profile was obtained by hierarchically clustering approximately 5,100 genes showing significant expression variation (standard deviation > 1.0) over all neurovascular cell types. Red and green colors indicate high and low gene expression levels, respectively, as indicated by the scale bar. (**b**) From the gene expression profile, we defined DEGs as genes showing an expression ratio above 2 or below 0.5 in each stimulated neurovascular cell type. Then, the effects of each herbal component were measured by observing the recovery of expression of DEGs. Recovered genes were defined as those whose expression was restored to normal levels (expression ratio between 0.5 and 2). The upper panel represents the distribution of recovered genes by each herbal component. The red and green color indicate up- and down-regulated DEGs, respectively, while the black color represents the position of genes recovered by individual herbal components. In order to compare the distribution of the recovered genes, the data are shown as a stepwise distribution diagram. The lower panel presents the number of DEGs and recovered genes by each herbal component. (**c**) Based on this recovery pattern of genes, we measured the number of genes recovered in common between two herbal components. After extending this measurement to all combinations of herbal components, we constructed the therapeutic network in terms of commonly recovered genes. Node size and edge thickness represent the number of recovered genes by each herbal component, and the number of commonly recovered genes between two herbal components, respectively. (**d**) The structure of the therapeutic network obtained from one neurovascular cell type was then compared with those from other cell types to obtain correlation patterns between neurovascular cell types.

We then measured the therapeutic profiles of UGS and its components on neurovascular cells based on gene expression. Figure 3a shows the overall patterns of gene expression in all experimental conditions. Because each type of stimulated neurovascular cells showed different patterns of gene expression, it would be hard to assess the therapeutic similarities between neurovascular cell types directly from expression levels. Instead, we examined the recovery ratio using restored genes (expression ratio between 0.5 and 2) among DEGs and compared the recovery patterns between neurovascular cell types by UGS and its components. The gene expression recovery profiles of each neurovascular cell type are shown in Fig. 3b. We confirmed that the genes recovered upon administration of UGS and its components were different in each neurovascular cell type, suggesting that the therapeutic effects of UGS might result from complex interactions among combination of genes induced by each individual herbal component. Using this recovery pattern of genes, we constructed the recovery network by herbal components. In detail, we measured the number of genes recovered in common between two herbal components. After iteration of this process to all pairs of herbal components, we constructed the therapeutic network based on the similarity of recovered genes (Fig. 3c). Node size represents the number of recovered genes by individual herbal components and edge thickness is proportional to the number of common genes between two herbal components.

Based on this therapeutic network structure, we evaluated the similarities among neurovascular cell types quantitatively, and represented them as correlation plots (Fig. 3d). Intriguingly, all pairs of neurovascular cell types showed significant correlation patterns (r > 0.5, p < 0.001) based on the therapeutic similarity of herbal components. In addition, we compared the therapeutic patterns of the mouse hippocampal tissue with those of neurovascular cells. Supplementary Fig. S7 shows that hippocampal tissue from Aβ-administered mice had high correlations with neurovascular cells (r > 0.6, p < 0.05), and especially with HT22 neuronal cells (r = 0.987, p < 0.001), although the number of herbal components used in the measurement of therapeutic patterns was small (only Mix1, Mix2, Mix3, and C7). Considering that HT22, BV-2, and HBVP cells together represent the neurovascular system, and that HT22 cells are of the same origin as mouse hippocampal tissue, this correlation pattern suggests that therapeutic profiles could represent biological features.

### Pathway activities by UGS and its herbal components

**Figure 4:**
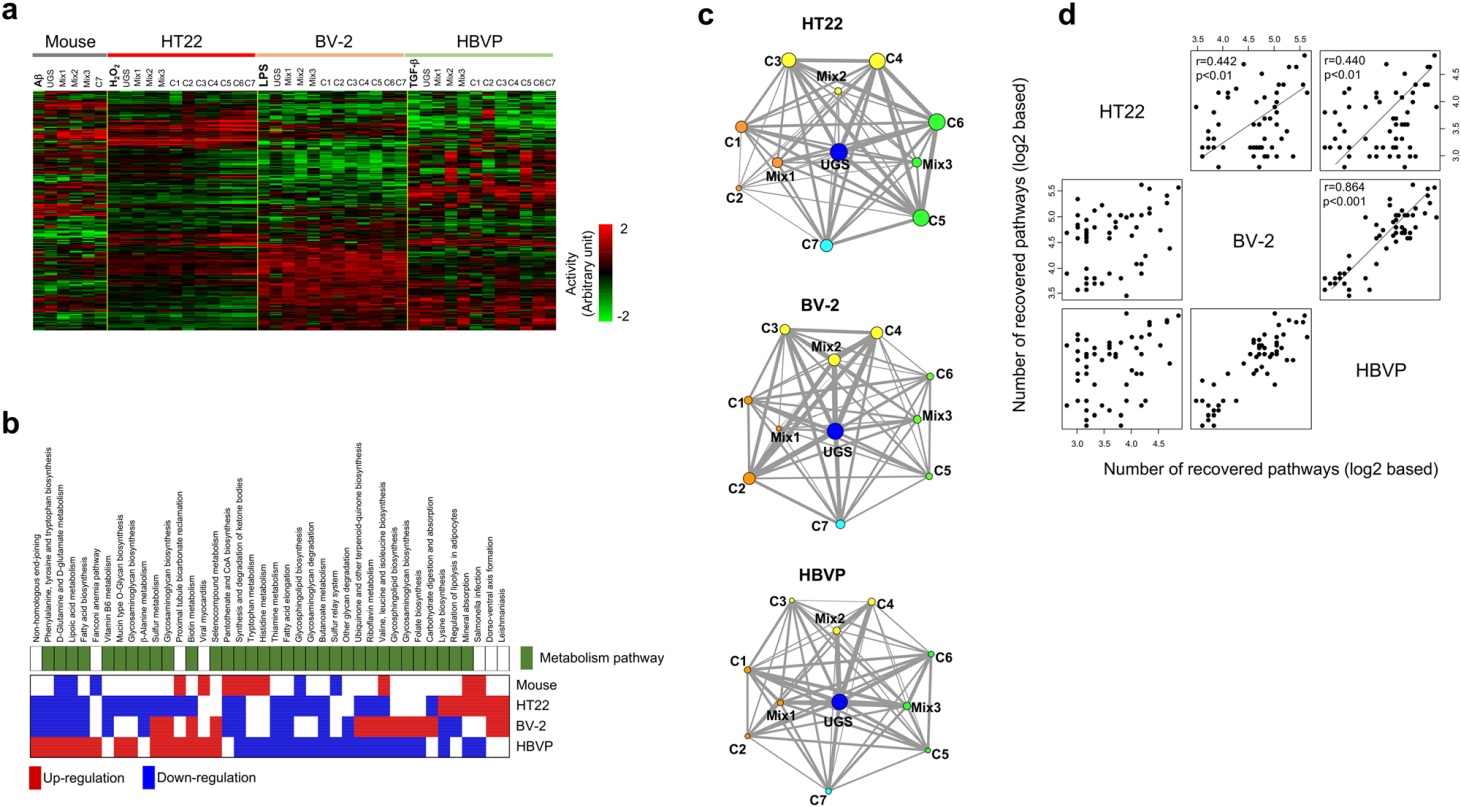
Therapeutic similarities between neurovascular cell types based on pathway activity. (**a**) Pathway activities from stimulated neurovascular cells were calculated by linearly combining expression levels of the genes included in each pathway. These values of pathways activities were then hierarchically clustered. Only pathways showing significant variations (FDR < 0.01) over all neurovascular cell types were included. The rows and columns represent individual samples pathways, respectively. The red and green colors reflect the high and low levels of pathway activity, respectively, as indicated by the scale bar in arbitrary units. (**b**) Distribution of pathways significantly involved (FDR < 0.001) among at least 2 types of stimulated neurovascular cells were examined. The position of metabolism-related pathways is indicated in green. Up- and downregulated pathways are colored in red and blue, respectively. (**c**) As in the case of gene expression, by measuring the therapeutic profile between each pair of herbal components in terms of commonly recovered pathways, therapeutic networks were constructed. Differentially regulated pathways were selected as those pathways with FDR ≤ 0.01 in stimulated neurovascular cells. Among these differentially regulated pathways, a 50% reduction in activity induced by herbal components was considered as recovery of the pathway activity. Based on the recovery pattern of pathway activity, we measured the number of pathways recovered in common between two herbal components. After extending this measurement to all combinations of herbal components, we constructed the therapeutic network in terms of commonly recovered pathways. Node size and edge thickness represent the number of recovered pathways by each herbal component and the number of commonly recovered pathways between two herbal components, respectively. (**d**) The structure of the therapeutic network obtained from each neurovascular cell type was then compared with those from other cell types to obtain the correlation patterns between neurovascular cell types.

In addition to gene expression, we used pathway activities to assess the therapeutic similarities among neurovascular cell types. Figure 4a shows the overall profile of pathway activities, in which we observed their different distribution depending on experimental conditions and neurovascular cell types. As expected from the functional connections shown in Fig. 2c–d, we confirmed the presence of common pathways between neurovascular cell types, with metabolism-related pathways in particular found to be commonly involved in at least two cell types. The most affected pathways (false discovery rate [FDR] < 0.001) in stimulated neurovascular cells are shown in Fig. 4b. However, the individual effects of UGS and its components on individual pathways were different, depending on cell type, as in the case of the individual genes shown in Fig. 3.

Therefore, rather than focusing on individual pathways, we measured the relationship between all pathway activities recovered by UGS and its components in each cell type (Fig. 4c). Based on these recovery network patterns, therapeutic similarities between neurovascular cell types were assessed by applying the same procedure previously used for gene expression (Fig. 4d). Neurovascular cells showed significant correlations with each other, with Pearson’s correlation coefficients greater than 0.4. In particular, BV-2 microglial cells showed high correlation with HBVP cells (r = 0.864, p < 0.001). We also measured the therapeutic patterns of hippocampal tissue from Aβ-administered mice, and compared them with those from neurovascular cells, as shown in Supplementary Fig. S8. Hippocampal tissue from Aβ-administered mice also showed significant correlations with neurovascular cells, and especially with HT22 neuronal cells (r = 0.692, p = 0.02), consistently with the results of the gene-based therapeutic similarity network shown in Supplementary Fig. S7.

### Phosphorylation regulations by UGS and its components

**Figure 5:**
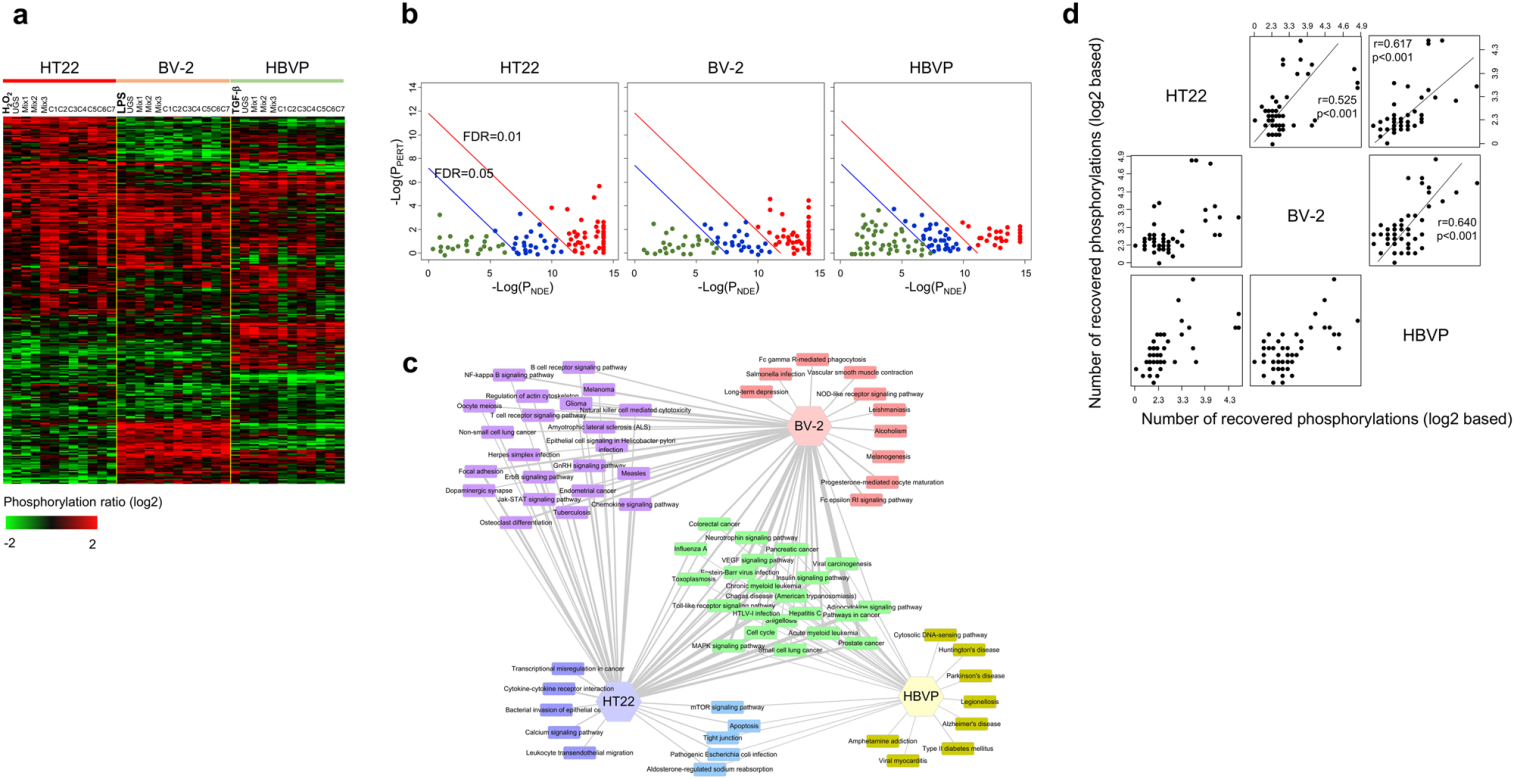
Regulation of phosphorylations by UGS and its components. (**a**) The phosphorylation status of signaling proteins was measured using antibody array technology from stimulated neurovascular cells. Protein phosphorylation levels were compared with those of control samples to obtain phosphorylation ratios. A phosphorylation profile was obtained by hierarchically clustering approximately 310 proteins showing significant variation (standard deviation > 1.0) over all neurovascular cell types. The red and green colors reflect high and low protein phosphorylation, respectively, as indicated by the scale bar. A detailed image is shown in Supplementary Fig. S9. (**b**) Enriched signaling pathways from protein phosphorylation in stimulated neurovascular cells were analyzed with the Signaling Pathway Impact Analysis (SPIA) program. The horizontal axis represents pathway overrepresentation (P_NDE_), while the vertical axis indicates pathway perturbation (P_PERT_). The red and blue circles on the right of the red oblique lines represent significant pathways after FDR correction (0.01 and 0.05, respectively) of the global p-values (P_G_), representing the pathway ranks calculated from the combination of both P_NDE_ and P_PERT_. The list of phospho-based pathways is shown in Supplementary Table S2. (**c**) The distribution of significant pathways (FDR < 0.01) from SPIA was displayed in connection with neurovascular cell types. A detailed image is shown in Supplementary Fig. S10. (**d**) From the phosphorylation profile, differentially phosphorylated proteins were defined as proteins showing a change of phosphorylation status above 2 or below 0.5 compared with control samples in each stimulated neurovascular cell type. Then, the effects of each herbal component were measured by observing recovery of phosphorylation. Recovered proteins were defined as those whose phosphorylation was restored to normal levels (phosphorylation ratio between 0.5 and 2). Based on the recovery pattern of phosphorylation, we measured the number of proteins recovered in common between two herbal components. After extending this measurement to all combinations of herbal components, we constructed the therapeutic network in terms of commonly recovered phosphoproteins. Then, the correlation between neurovascular cell types was assessed in terms of the similarity of patterns of phosphorylation recovery by herbal components.

The Gene Ontology analysis shown in Fig. 2 shows that metabolism and signaling-related biological functions were both involved in the stimulation of neurovascular cells. Since we had revealed the importance of metabolism-related pathways as commonly associated functions in all types of neurovascular cells (Fig. 4b), we then focused on signaling events, by measuring the phosphorylation status of diverse signaling proteins. Figure 5a presents the profile of proteins phosphorylation status in neurovascular cells stimulated by each experimental condition. A detailed image is shown in Supplementary Fig. S9. Overall differences depending on the experimental condition were assessed in the phosphorylation status of signaling proteins.

We then identified significantly enriched signaling pathways (FDR < 0.05) in stimulated neurovascular cells based on the phosphorylation levels of signaling proteins (Fig. 5b). The list of enriched pathways is shown in Supplementary Table S2. The distribution of these enriched phospho-based signaling pathways demonstrated the presence of common as well as cell-specific pathways in stimulated neurovascular cells (Fig. 5c; a detailed image is shown in Supplementary Fig. S10). Moreover, we observed a significant correlation (r > 0.3, p < 0.01) in the FDR distribution of the commonly enriched phospho-based pathways (FDR < 0.01) among all neurovascular cells (Supplementary Fig. S11). These correlated patterns of FDR distribution were also observed between samples treated with each herbal component (Supplementary Fig. S12). Therefore, it is difficult to detect therapeutic differences among herbal components in terms of FDR distribution, although functional similarity could be verified. The quantitative correlation patterns between neurovascular cells based on the FDR distribution of each herbal component are shown in detail in Supplementary Fig. S13.

Therefore, we measured the therapeutic network in terms of the signaling protein phosphorylation recovery patterns induced by the treatment with UGS and its components in each neurovascular cell type, then examined the therapeutic similarities between neurovascular cells, as in previous sections. Figure 5d shows that all 3 neurovascular cell types showed significantly high correlations, with correlation coefficients >0.5, the highest correlation being between BV-2 microglia and HBVP pericytes (r = 0.640, p < 0.001), in agreement with the results from gene expression (Fig. 3d) and pathway activity (Fig. 4d).

### Network analysis

**Figure 6:**
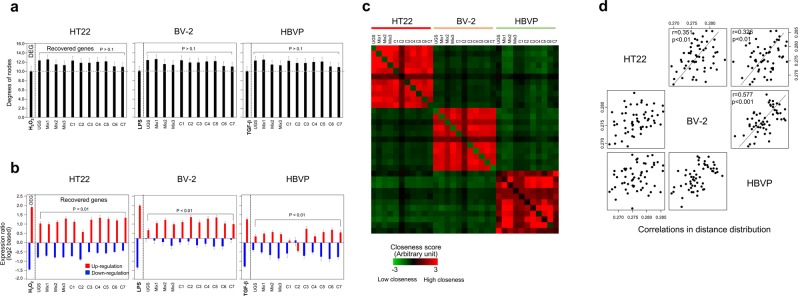
Therapeutic effects of UGS and its components in the PPI network. (**a**) The network degree distribution of the genes recovered by treatment with UGS and its components was examined in each stimulated neurovascular cell type. The 2-fold criterion was applied to identify DEGs and recovered genes, as described in the legend to Fig. 3. Network degree values were obtained by mapping the genes onto the human PPI network. The error bar indicates the standard error. Statistical difference on degree values between DEGs and recovered genes was measured using t-test. (**b**) Expression changes due to treatment with herbal components were measured in major gene modules. Modules were identified with the Reactome FI Cytoscape plugin program using DEGs from each neurovascular cell type. Expression levels are represented in red and blue for up- and downregulated genes, respectively. The effects of UGS and its components on the expression level of the genes included in each module were then evaluated. The error bar indicates the standard error. Statistical difference on expression values between DEGs and recovered genes was measured using t-test. (**c**) Distances between genes recovered by UGS and its components were measured in terms of the shortest path length in the PPI network. Red and green colors represent high and low closeness in the PPI network, respectively. (**d**) Based on the shortest distances in PPI network among recovered genes by herbal components, we constructed the therapeutic network. Then, the correlation between neurovascular cell types was assessed in terms of similarity of the patterns of distance distribution among the genes recovered by herbal components.

In the preceding sections, genes or their associated biological functions such as pathways were directly used to measure the therapeutic effectiveness of herbal components or the therapeutic relationship between neurovascular cell types. In this section, we used a novel approach based on the topological characteristics of the relevant genes in a PPI network. Figure 6a shows that the genes restored by the treatment with UGS and its components had higher network degree than the DEGs in stimulated neurovascular cells, although the statistical significance of these differences was marginal or non-significant (p > 0.1). We then focused on a group of co-regulated genes, considered as a module, because herbal drugs could possibly exert their therapeutic effects through multiple target genes. Supplementary Fig. S14 represents the core modules isolated from each stimulated neurovascular cell type. The therapeutic effects of herbal components were confirmed in terms of core modules, as shown in Fig. 6b. UGS and all herbal components recovered stimulus-induced up- or down-regulated core modules in each neurovascular cell type (p < 0.01).

As another parameter derived from network analysis, we measured the shortest distance in the PPI network between genes restored by each herbal component. As expected, restored genes by herbal components from one cell type were more closely distributed among each other than with those from other cell types (Fig. 6c). However, when we compared the patterns of the distance distribution of the recovered genes by herbal components among cell types, significant correlations were observed between neurovascular cell types, with correlation coefficients greater than 0.3 (p < 0.01), as shown in Fig. 6d. Consistently with previous results, BV-2 microglial cells and HBVP pericytes showed the highest correlation (r = 0.577, p < 0.001).

### Connectivity map

As a final step, we used the Connectivity Map to verify whether therapeutic profile similarity measurements could provide biological information about the therapeutic properties of a drug^44^. Note that since chemical compounds were individually administered to each cell type, the experimental conditions of the Connectivity Map are different from our own. Among the chemical compounds with available data, we focused on kinase inhibitors, the category including the largest numbers of compounds (306). Kinase inhibitors were classified into 3 major categories (cell cycle inhibitors, inhibitors of growth factor receptors, and mitogen-activated protein kinase [MAPK] inhibitors) and more detailed functional subcategories as described in the Materials and methods (see Supplementary Table S3).

**Figure 7:**
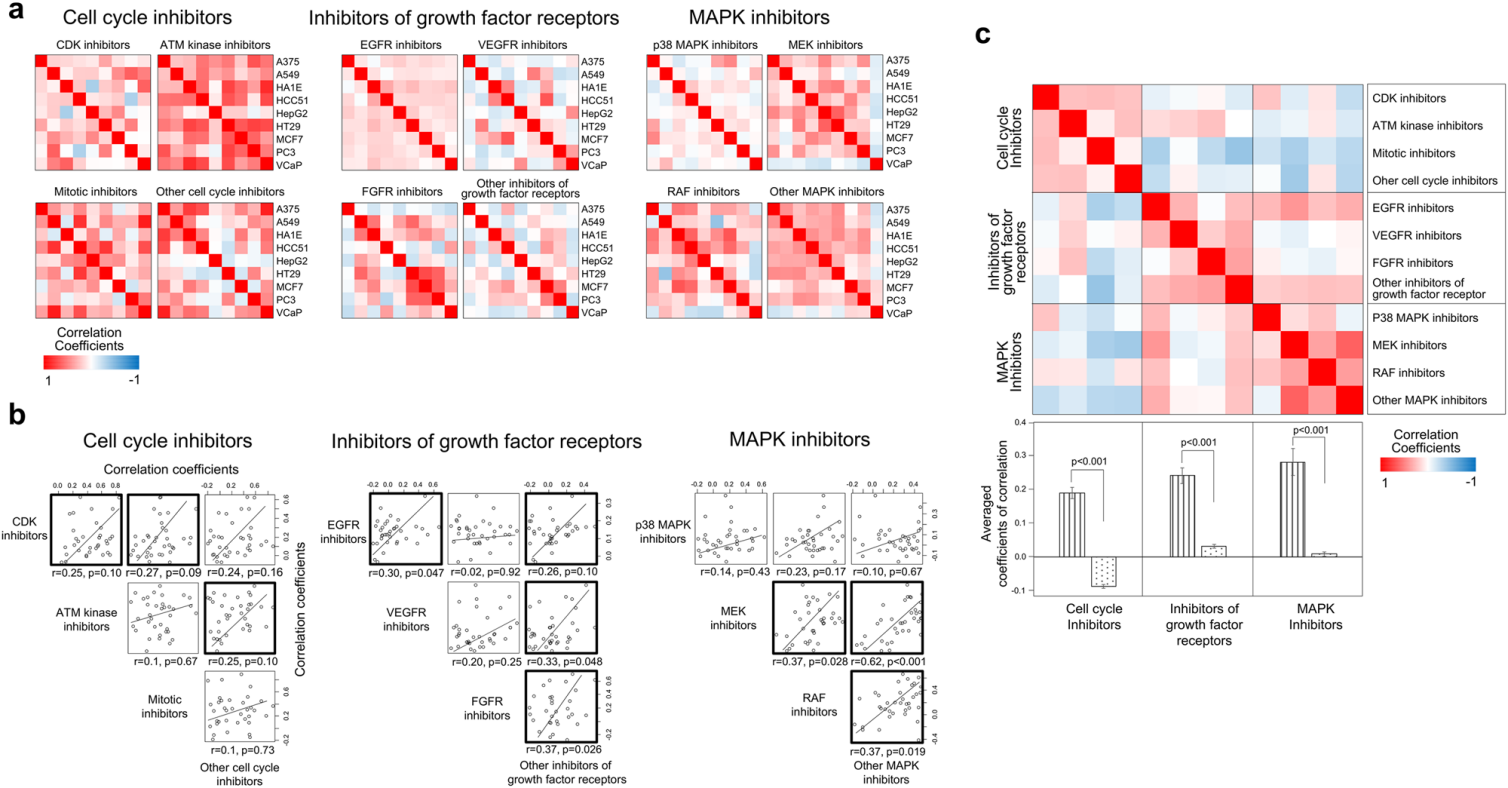
Therapeutic similarity in the Connectivity Map. Information on the expression of genes responding to kinase inhibitors was obtained from the Connectivity Map database. (**a**) Based on the expression patterns of genes responding to kinase inhibitors, the therapeutic similarity between nine types of cancer cells (A375, A549, HA1E, HCC51, HepG2, HT29, MCF7, PC3, and VCaP cells) was measured. Kinase inhibitors were classified into 3 major categories: cell cycle inhibitors, inhibitors of growth factor receptors, and MAPK inhibitors. Then, each of these major categories was further subdivided into four functional subcategories. By measuring the response similarities between all possible pairs of cell types to each kinase inhibitor included in the functional subcategories, correlations between cell types were obtained. (**b**) Using such cell type similarity profiles, correlations among functional subcategories were then evaluated. Pairs of functional subcategories showing significant (p < 0.05) or marginally significant (p < 0.1) correlations are emphasized with bold lines. (**c**) The relationships between the major categories were assessed based on the subcategory correlation pattern. In the lower panel, the striped and dotted bars represent the average correlation coefficients within and among major categories, respectively. The error bar indicates the standard error. Statistical difference on coefficients values was measured using t-test.

We computed the correlation profiles among nine cell types based on the gene expression response to the chemical compounds included in each functional subcategory (Fig. 7a). At least 10 pairs of chemical compounds included in each functional subcategory were compared with each other to yield correlation coefficient values for each cell type, and we then compared the response similarities between functional subcategories. As shown in Fig. 7b, at least 50% of the pairs of functional subcategories in each major categories showed significant (p < 0.05) or marginally significant (p < 0.1) correlations, with coefficients above 0.25. In addition, we also computed correlations across functional categories: Fig. 7c shows that kinase inhibitors within the same category were more closely correlated than with inhibitors belonging to other categories. The distribution of the correlation coefficients clearly indicates the segregation of kinase inhibitors depending on the biological features of their targets (p < 0.001). These results imply that our approach based on therapeutic similarity of drugs on diverse cell types could indeed provide information on the molecular mechanisms of chemical actions.

## Discussion

We proved that UGS and/or its components protected neurovascular cells against diverse damaging agents and improved cognitive functions in an animal model. In addition to our results, individual herbal components have been reported to have various biological activities including neuroprotective, anti-inflammatory, and/or antioxidant activities, as shown in Supplementary Table S1. These versatile therapeutic effects of UGS or its herbal components in diverse experimental conditions imply that UGS could exert its therapeutic effects through various biological processes. Since the occurrence and progression of AD result from diverse causes affecting various types of cells in the brain, drugs such as UGS with multi-targeting ability could be a promising therapeutic choice.

Previously, we identified a few representative chemical compounds included in UGS using the analytical profile of high-performance liquid chromatography (HPLC) in which decursin, glycyrrhizin, and decursinol angelate were predominantly included^18^. Moreover, one of them, namely ferulic acid, shows antioxidant activity. However, such chemical compound-based approach has its limitation because we could not identify all active chemical compounds from UGS, and moreover a few isolated chemical compounds could not represent the whole therapeutic activity of UGS. Therefore we hypothesized that an approach using whole extracts of this herbal drug could give novel therapeutic information that could not be obtained when using individual chemical compounds.

*In vivo* and some *in vitro* experiments presented in our study show that therapeutic effects were more prominent when individual components (C1–C7) or mix groups (Mix1–Mix3) were administered rather than UGS itself. This could presumably represent counteractive interactions among the 7 herbal components in the UGS therapeutic action. However the therapeutic efficacies of herbal components should not be directly compared with each other in parallel. Since treatment concentrations were determined as the doses showing maximal therapeutic effect without toxicity in each experimental condition, the actual treatment concentrations were different even within the same herbal component.

Moreover, depending on cell type, each stimulus induced different gene expressions, pathway activities, kinase phosphorylation, etc. Therefore, a direct comparison of the therapeutic efficacy of individual herbal components between cell types might be difficult. Instead, we introduced a novel approach—therapeutic similarity measurement—in which the therapeutic relationships among all possible combinations of herbal components measured in one type of cells were compared with those measured in the other types of cells. In this approach, the therapeutic relationship between herbal components was measured using the number of recovered genes, number of recovered pathways, etc. Since we applied therapeutic similarity measurement, we speculate that the difference in experimental conditions between cell types could not significantly influence the comparisons. In other words, we focused on the therapeutic patterns rather than the therapeutic effectiveness of individual components and observed the similarity among neurovascular cell types in such therapeutic patterns exerted by UGS and its components.

The results of the present study consistently indicate significant correlations between neurovascular cells (HT22, BV-2, and HBVP) in terms of the therapeutic patterns induced by herbal components, BV-2 microglia and HBVP cells showing the highest similarity. Considering that microglia and pericytes play different roles in the brain, our results could imply biological similarities between these two types of cells. Interestingly, recent publications have reported a close relationship between microglia and pericytes in the brain. For example, pericytes can mediate neuroinflammation by recruiting leukocytes, activating microglia, and inducing endocytosis^45^. Moreover, pericytes can acquire a microglial phenotype^46^ or multipotent vascular stem cell activity to serve as a source of microglia^47^. In addition, the importance of cellular communication between neuronal cells and vascular cells in the brain has been already underlined as relevant in the occurrence and progression of AD^48,49^.

Likewise, similar therapeutic patterns in diverse experimental conditions suggest underlying similarities in the pharmacological action by herbal components between neurovascular cell types. Moreover, the high correlation in the patterns of distance distribution in the PPI network further supports the possible presence of underlying fundamental similarities between neurovascular cells, when considering that closeness in PPI network represents closeness in biological functions, as we have previously shown^50^.

Interestingly, the biological responses resulting from diverse experimental conditions in the present study were connected to each other to form a huge network of biological functions. In addition, it has been reported that the administration of Aβ in the hippocampus of mice increases the oxidative stress in the brain through inflammatory cytokines such as TNF-α^14^. We thus speculate that the similarities of therapeutic profiles could result from this biological aspect common among neurovascular cells. This view is also confirmed by the observation that the hippocampus of Aβ-administered mice showed a therapeutic profile similar to that of H_2_O_2_-treated HT22 neuronal cells (Supplementary Fig. S7). These two cell types indeed share a common biological feature, originating from hippocampal cells. In this study, we used whole hippocampal mouse tissue, where there exist several cell types. Although we did not isolate or quantify the number of each type of neurovascular cells contained in hippocampal tissues, we assume that neurons were the main type of cells, considering that neuronal markers such as *Eno2*^51^, *Rbfox3* (NeuN)^52^, *Map2*^53^, and *Tubb3*^54^ were highly expressed when compared with markers of pericytes and activated microglia (Supplementary Fig. S15).

Finally, we examined whether our approach of therapeutic similarity could be used to find biological meaning using the Connectivity Map. Although this public database uses single chemical compounds, not herbal extracts, individually treated to each cancer cell type, we successfully segregated functionally correlated chemical compounds based on similarity measurement of therapeutic (or expression) patterns by cell type. These results demonstrated that the measurement of therapeutic patterns could give valuable biological information on the molecular mechanism of UGS on neurovascular cells. However, this does not imply the existence of a single common factor behind the action of the drug on neurovascular cells, considering that the cellular responses to external stimulants were different depending on the cell type and the stimulant. Rather, we surmised that therapeutic patterns could be used as a novel parameter able to capture hidden information about the therapeutic properties of UGS in diverse neurovascular cells.

Our approach based on therapeutic similarity of herbal drugs could also be applied to any drug composed of multiple chemical components. In order for our approach to be more systemized and applicable to other multi-component drugs, the following aspects should be further confirmed. First, therapeutic effectiveness for as many as possible drug components should be measured and compared in a larger number of cell types and pathological conditions. By increasing the number of experimental conditions in this way, it is expected to observe more robust therapeutic relationships between components and between cell types. As a pilot study, we used only 10 different herbal combinations, consisting of 7 individual components and 3 mixture groups, to measure the therapeutic profiles. Each mixture group was composed of 2 herbal components showing neuroprotective or anti-neuroinflammatory activity. However, this is a small part of the total number of herbal combinations that can be created out of 7 different components. Therefore, by measuring the therapeutic profiles of a larger sample of herbal combinations, we could obtain more robust therapeutic information. Second, the biological meaning of the similarity in therapeutic profiles between diverse cell types should be interpreted in terms of detailed molecular mechanisms. Although we suggested that therapeutic profiles could be associated with cellular origin or drug molecular targets, this assumption should be verified in diverse pathological conditions with diverse cell types.

In the present study, we focused on the measurement of multiple rather than individual genes to examine the therapeutic effects of UGS on neurovascular cells stimulated by various damaging agents. This is not only because herbal medicines such as UGS can regulate multiple gene targets involving diverse functions, but also because measurement of therapeutic patterns is insensitive to inherent errors in measuring expression values of individual genes. However, at the individual gene level, complementary or synergistic interactions can occur by complex interactions between the herbal components and their target genes. Consequently, genes induced by one component could be suppressed by another component. Therefore, to fully understand the therapeutic effects of herbal drugs, it is necessary to integrate similarity with complementarity in the therapeutic actions exerted by their components at the individual gene levels.

In conclusion, UGS and/or its herbal components protected neurovascular cells *in vitro* against diverse damaging agents and improved cognitive functions in an Aβ-induced mouse model of AD. We observed similarity among neurovascular cell types in terms of the therapeutic patterns exerted by UGS and its components. These findings, together with the functional connections between neurovascular cell types, can contribute to our understanding of the therapeutic effects of UGS.

## Materials and Methods

### Plant materials and preparation of ethanol extracts of herbal medicines

Seven crude extracts forming UGS, namely Uncariae Ramulus et Uncus (C1), Atractylodis Rhizoma Alba (C2), Poria Sclerotium (C3), Bupleuri Radix (C4), Angelicae Gigantis Radix (C5), Cnidii Rhizoma (C6), and Glycyrrhizae Radix et Rhizoma (C7) were obtained from Kwang-myung-dang Pharmaceutical Co. Ltd. (Ulsan, Republic of Korea). These seven crude extracts were mixed as indicated in Table 1 to prepare UGS.

In the preparation of mixture groups (Mix1–Mix3), the ratios of the two herbal extracts reproduced those contained in UGS. To prepare Mix1, C1 and C2 were mixed in the ratio 3:4. For Mix 2, C3 and C4 were mixed in the ratio 2:1. For Mix 3, C5 and C6 and were mixed in the ratio 1:1.

The prepared mixed or single crude herbs were extracted using 70% aqueous ethanol by refluxing at 100 °C for 2 h. The extract was then filtered through a paper filter with a pore diameter of 5 μm and concentrated using a vacuum rotary evaporator system (EYELA N-1000, Bohemia, NY, USA) to give a powder, as described in our previous paper^18^. Voucher specimens have been deposited in extract bank at Korea Institute of Oriental Medicine.

### Cell culture

HT22 mouse hippocampal cells and BV-2 mouse microglia were cultured in Dulbecco’s Modified Eagles medium (Thermo Fisher Scientific, Rockford, IL, USA), supplemented with 10% fetal bovine serum (FBS; Thermo Fisher Scientific) and 1% penicillin/streptomycin in a 5% CO_2_ at 37 °C. HBVP human brain vascular pericytes, were obtained from ScienCell Research Laboratories (Carlsbad, CA, USA) and incubated in pericyte medium (ScienCell Research Laboratories) supplemented with 2% FBS, 1% pericyte growth supplement, and 1% penicillin/streptomycin in a 5% CO_2_ at 37 °C.

### Cytotoxicity test

The neuroprotective effects of UGS and its herbal components against hydrogen peroxide (H_2_O_2_) was measured using a cell proliferation kit (CCK) (Dojindo, Kumamoto, Japan). Briefly, after plating HT22 cells (5 × 10^3^/well) on 96-well microplates, H_2_O_2_ (250 μM) and each herbal extract were treated for 6 h. Then CCK-8 solution was added, and the cells were further incubated for 4 h. The cell viability was calculated by measuring optical density (OD) at 450 nm on an Epoch Microplate Spectrophotometer (BioTek, Winooski, VT, USA) according to manufacturer instructions.

### Enzyme-linked immunosorbent assay (ELISA) for TNF-α

The quantity of TNF-α was measured using a Mouse TNF-α Quantikine ELISA kit purchased from R&D systems (Minneapolis, MN, USA). After pretreatment with each herbal extract for 2 h, BV-2 cells were then stimulated with LPS (1 μ/mL) for an additional 22 h. Cell culture supernatants were transferred to 96-well microplates coated with mouse anti-TNF-α antibody and then incubated with TNF-α conjugate. The concentration of TNF-α in each well was calculated by comparison with the standard concentrations provided in the kit according to manufacturer instructions.

### Gelatin zymographic assay for MMP-9

MMP-9 enzymatic activity was assessed by using gelatin zymography. HBVP cells were plated onto 6-well plates and kept to 90% confluence in 2 ml of culture media. Cells were treated with TGF-β1 (10 ng/mL) and each herbal extract for 24 h, and the supernatants were collected. After measuring protein concentrations in the supernatants, equal amounts of samples were mixed with tris-glycine SDS buffer for 15 min and then electrophoresed onto the Novex Zymogram Plus gelatin protein gels (Thermo Fisher Scientific, Rockford, IL, USA). After renaturation of gels in the renaturing buffer (Thermo Fisher Scientific) for 45 min and development in the developing buffer at 37 °C overnight, the gels were stained for 1 h with 0.5% Coomassie brilliant blue R-250 solution (Thermo Fisher Scientific) containing 10% acetic acid and 20% methanol, and then destained for 4 h in solution with 7.5% acetic acid and 5% methanol. Bands of MMP-9 (molecular weight = 92 kDa) were visualized using the ChemiDoc Gel Imaging System (Hercules, CA, USA).

### Animals

ICR mice (male, 8-week-old) were obtained from Dae Han Biolink Co., LTD. (Eumseong, Republic of Korea). Mice were housed in a pathogen-free room with a controlled temperature (22 °C ± 3 °C) and a 12 h light/12 h dark cycle. Standard food pellets and drinking water were provided *ad libitum*. Every effort was made to minimize pain in the animals during the experiment. All experimental procedures were approved by the Institutional Animal Care and Use Committee of the Korea Institute of Oriental Medicine (Approval number: #17–034) and carried out in accordance with the provisions of the National Animal Welfare Law of Korea and the Guide for the Care and Use of Laboratory Animals of the National Research Council (US) Institute for Laboratory Animal Research.

### Injection of Aβ_1–42_ aggregates

Injection of Aβ_1–42_ aggregates into the mouse brain followed the methods of our paper previously published^50^. Mice were acclimated at the animal care facility 1 week prior to injection of Aβ_1–42_ aggregates. Aβ_1–42_ peptides (AnaSpec, Fremont, MO, USA) were dissolved in phosphate-buffered saline (PBS) to the final concentration of 1 mM. Aβ_1–42_ aggregates were then generated by incubation at 37 °C for 7 days. Aβ_1–42_ aggregates (10 μmol) were then injected into the intracerebroventricular (ICV) region of the brain of mouse positioned in a stereotaxic frame. For midline sagittal incision, the skull was punctured over the lateral ventricle using the following stereotactic coordinates: –0.5 mm anteroposterior; 1 mm mediolateral; and 2.5 mm dorsoventral. A Hamilton syringe with a 26-gauge stainless-steel needle was used to deliver Aβ_1–42_ aggregates at a rate of 1 μL/min. During the process of injection, body temperature of mouse was maintained at 36.5 ± 0.5 °C. The same procedure was applied to the control animals except that the same amount of vehicle was injected. Daily oral administration of vehicle or each herbal extract was continued for 21 days.

### Passive avoidance task

The passive avoidance task tests were conducted as described in our previous report^55^. We used an apparatus (Jungdo Bio, Kwangju, Republic of Korea) consisting of two equally sized chambers (40 cm × 20 cm × 30 cm) separated by a guillotine door in the middle. One chamber was illuminated with a 60 W light bulb and the other chamber was kept dark. The bottom of the dark compartment was made of 5 mm stainless steel bars at 1 cm intervals. Mice were orally administered with saline (vehicle) or herbal extracts (UGS, Mix1‒Mix3, and C7) daily for 21 days. On day 14 after injection of Aβ_1–42_ aggregates, the animals were individually placed into the lit chamber and allowed to freely explore it for the training. After 20 seconds, the guillotine door was lifted to allow the animal to enter the dark compartment. On day 15, the same procedure was repeated but this time an electric shock lasting 3 seconds at 0.5 mA was delivered to the mice through the grid floor immediately after it entered the dark compartment. Tests were performed on day 16 after injection of Aβ_1–42_ aggregates by measuring the latency spent in the light compartment before entering the dark compartment for up to 300 s.

### Immunohistochemistry (IHC)

Immediately after anesthetization, mice were perfused with PBS to wash the vascular system and then fixed with cold 4% paraformaldehyde (PFA) solution. The brains were removed and fixed in PFA solution (4%) overnight at 4 °C. Using a freezing microtome (Leica Biosystems, Nussloch, Germany), brains were cut into frozen sections with 30 μm-thick. For IHC analysis, these brain sections were treated with 1% H_2_O_2_ diluted in PBS for 15 min and then incubated overnight at 4 °C with the primary antibodies (1:1000) including anti-NeuN (Merck Millipore, Darmstadt, Germany), anti-Iba-1 (Wako Pure Chemical Corporation, Richmond, VA, USA), or anti-desmin (Abcam, Cambridge, UK). After rinsing with PBS, the sections were then incubated for 1 h with biotinylated anti-rabbit immunoglobulin G (1:500), washed, and incubated with Avidin–Biotin Complex (1:100) for 1 h at room temperature. The signals from the peroxidase activities were visualized using 3,3′-diaminobencidine in 0.05 M Tris-buffered saline (pH 7.6). The brain sections were washed with PBS and mounted on gelatin coated slides. Quantitation of histological staining was performed using ImageJ^56^.

### Statistical analysis of *in vivo* and *in vitro* experiments

The *in vivo* and *in vitro* experimental data are expressed as the mean ± standard error. Statistical analysis was conducted using one-way analysis of variance with Dunnett’s test for multiple comparisons using GraphPad Prims 6.0 (San Diego, CA, USA).

### RNA isolation

Total RNAs were purified from each cell line and whole hippocampal mouse tissue using Trizol according to manufacturer instructions (Thermo Fisher Scientific, Rockford, IL, USA). The quality of the RNA was assessed by a 2100 Bioanalyzer Instrument (Agilent, Santa Clara, CA, USA). We included only samples in the QuantSeq analysis with an RNA integrity number >7.0.

### Library preparation and sequencing for QuantSeq analysis

The double-stranded cDNA library for sequencing was prepared using QuantSeq. 3′ mRNA-Seq Library Prep Kit (Lexogen, Vienna, Austria) according to manufacturer instructions. In brief, from 500 ng total RNA, first strand of cDNA was generated using reverse transcription with oligo-dT primer containing an sequencing tag at its 5′ end. Second strand was then synthesized by a random primer containing an linker sequence tag at its 5′ end. After removal of all reaction components, the cDNA library was amplified by polymerase chain reaction with adapters. The finished library was purified using purification beads and then subjected to sequencing using Illumina NextSeq. 500 system (Illumina, CA, USA) to obtain 75 base pair (bp) single-end reads.

### QuantSeq data analysis

Aligning QuantSeq reads to a reference genome (genome assembly version of mm10 for mouse and hg19 for human) was performed using Bowtie2^57^. From the generated alignment file, the amount of transcripts was estimated. Read count data were then quantile normalized using the edgeR R package^58^. Genes with expression ratios above 2 fold or below 0.5 in each experimental condition compared with control samples were considered differentially expressed genes (DEGs). Among these DEGs, those with expression ratio between 0.5 and 2 upon treatment with drugs were considered as restored by the treatment. Genes were hierarchically clustered using the Gene Cluster 3.0^59^. Visualization as a heatmap was performed using Java TreeView program^60^. Information on the correspondence between mouse and human gene symbols was obtained from the Jackson Laboratory’s orthology database (http://www.informatics.jax.org).

### Functional network of GO terms

Connections among GO terms as a functional network structure were measured using the ClueGO Cytoscape plugin program^41^. DEGs from each experimental condition were queried into ClueGO by applying the default parameter settings including FDR < 0.01.

### Enrichment map

The relationship between GO terms and gene sets was established using the Enrichment Map (v2.1.0) Cytoscape plugin application^42^. GO terms (FDR < 0.01) associated with DEGs from each stimulated neurovascular cell type were identified using DAVID^61^ and redundancy removed using ReviGO^43^. The sets of DEGs were connected with each GO term using a cutoff of 0.01 on the hypergeometric test.

### Measurement of similarity between therapeutic profiles

Similarities between neurovascular cells were measured based on experimental parameters such as the number of recovered genes, the number of recovered pathways, or the shortest distances between genes in the protein-protein interaction (PPI) network. For each neurovascular cell type, the therapeutic effects of each herbal component on these experimental parameters were measured. Then, the therapeutic effects were compared between two herbal components in terms of the number of common recovered genes and pathways, and the shortest distance in the PPI network between recovered genes. This process was iterated for all pairs of herbal components, and the values obtained from one type of neurovascular cells were compared with those from other cell types. Finally, the Pearson correlation coefficient was used as a therapeutic profile similarity index between two types of neurovascular cells.

### Pathway activity

As a measure of pathway activity, the expression values of genes included in the pathway were used as previously reported by us^29,30,50^. In brief, for each pathway, the log-transformed values of expression ratios of genes compared to the control were added linearly. The genes corresponding to suppressors were given a weight of −1. Repressors were defined as proteins that inhibit the pathway signal transduction. Statistical significance for the measured value of pathway activity was calculated by measuring permutation p-value (n = 1,000). Pathway information such as included genes and signal transduction directions is defined in the Kyoto Encyclopedia of Genes and Genomes database (KEGG, http://www.genome.jp/kegg/).

### Antibody array

Antibody microarray experiments were performed using the Phospho Explorer Antibody Array from Full Moon Biosystems (Full Moon Biosystems, Sunnyvale, CA, USA) according to manufacturer instructions. Briefly, the protein was extracted using a protein extraction buffer and the concentration of purified protein was measured with the BCA protein assay kit (Thermo Fisher Scientific, Rockford, IL, USA) using NanoPhotometer (Implen, München, Germany). A 50-μg protein sample was labeled with biotin and then hybridized with the microarray slide for 2 h at room temperature. After washing the slide, Cy3-streptavidin (GE Healthcare Bio-Sciences, Pittsburgh, PA, USA) in detection buffer was mixed with the array slide for 20 min. The slide was washed and then scanned using the GenePix 4100 A Microarray scanner (Molecular Devices, San Jose, CA, USA). Images were quantified with GenePix Pro 7.0 Software (Molecular Device) and analyzed using Genowiz 4.0 (Ocimum Biosolutions, India) to obtain the level of phosphorylation. Information on protein annotation was obtained from the UniProt database (http://www.uniprot.org).

### Signaling Pathway Impact Analysis (SPIA)

As another systematic pathway analysis, Signaling Pathway Impact Analysis (SPIA) was performed, which uses the topology of DEGs to calculate index for signaling pathway^62^. Specifically, two statistical measurements, P_NDE_ and P_PERT_, representing respectively the over-representation of input DEGs in a given pathway and the abnormal perturbation of that pathway, were calculated using 3,000 bootstrap iterations. Then, the global p-value (P_G_) was calculated for each pathway by combining P_NDE_ and P_PERT_ to select the significant pathways.

### Modules in network analysis

The functional network of genes was constructed using the ReactomeFIPlugIn Cytoscape application^63^. Specifically, a subset of DEGs was queried into Reactome FI data source to generate network modules composed of correlated genes based on information such as PPIs, gene coexpression, and so on. Identification of network modules was performed using the Markov Cluster Algorithm with a cutoff on Pearson’s correlation coefficients of 0.8 and default settings for the other parameters.

### Characteristics of the PPI network

The distance measurement between sets of genes on the network followed the method of our previous paper^50^. In brief, after mapping genes onto the PPI network obtained from BioGRID (version 3.4)^64^, the shortest path length between two genes was measured using the igraph R package (release version 1.1.1)^65^. By applying this measurement to all gene pairs in two gene sets (or two pathways), a matrix of shortest paths length was produced. The average value of the matrix of shortest paths length was interpreted as the distance index between two sets of genes (or two pathways). Only gene sets with more than >50% of the genes included in the PPI network were included. The distance index matrix was clustered and visualized using Cluster 3.0^59^ and Java TreeView program^60^, respectively.

### Connectivity Map analysis

Information on changes in gene expression induced by chemical compounds was obtained from the Touchstone dataset in the Connectivity Map^44^. This dataset has been obtained from a set of approximately 8,000 perturbagens, including chemical compounds (2,429), gene knock-downs (3,799), and gene overexpression (2,160) on 9 cell lines (A375, A549, HA1E, HCC51, HepG2, HT29, MCF7, PC3, and VCaP cells). Among these perturbagens, we selected the compounds acting as kinase inhibitors, for a total of 306 chemicals. Based on the annotated biological functions provided in the Connectivity Map, kinase inhibitors were divided into 3 major categories, namely cell cycle inhibitors, inhibitors of growth factor receptors, and mitogen-activated protein kinase (MAPK) inhibitors. Each major category was then classified into functional subcategories which included at least 5 individual chemical inhibitors administered to all cell types. For example, the category of cell cycle inhibitors included functional subcategories such as cyclin-dependent kinase (CDK) inhibitors (10 compounds), Ataxia telangiectasia mutated (ATM) kinase inhibitors (5 compounds), mitotic inhibitors (5 compounds), and other cell cycle inhibitors (5 compounds). Inhibitors of growth factor receptors were classified into epidermal growth factor receptor (EGFR) inhibitors (35 compounds), vascular endothelial growth factor receptor (VEGFR) inhibitors (7 compounds), fibroblast growth factor receptor (FGFR) inhibitors (6 compounds), and other inhibitors of growth factor receptors (5 compounds). The MAPK inhibitors included p38 inhibitors (12 compounds), MEK inhibitors (6 compounds), RAF inhibitors (6 compounds), and other MAPK inhibitors (5 compounds). The list of individual chemical compounds included in each functional subcategory of kinase inhibitors is shown in Supplementary Table S3.

The relationship between different cell types was assessed based on the similarity in gene expression changes in response to the chemical compounds included in each functional subcategory. For each cell type, the expression response to two chemical compounds was compared using the correlation coefficient, and this process was iterated for all pairs of chemical compounds. Then the correlation coefficients obtained from one type of cell were compared with those from other cell types. Finally Pearson’s correlation coefficients were computed as a similarity index in therapeutic profiles between two cell types. The values of the similarity index measured in all pairs of cells for each functional subcategory were compared with those of the other functional subcategories. By comparing all pairs of functional subcategories, we obtained the correlation coefficients representing the similarity of functional subcategories.

### Ethics approval

All experimental procedures were conducted in accordance with the National Institutes of Health Guidelines for the Care and Use of Laboratory Animals and were approved by the Institutional Animal Care and Use Committee of the Korea Institute of Oriental Medicine (Approval number: #17–034). Animal handling was carried out in accordance with the dictates of the National Animal Welfare Law of Korea.

## Supplementary information

Supplementary Information.

## Data Availability

Gene expression data information is registered on the Gene Expression Omnibus (GEO) site (http://www.ncbi.nlm.nih.gov/geo) with accession number GSE134414.
