# Supplementary material for: Similarity of therapeutic networks induced by a multi-component herbal remedy, Ukgansan, in neurovascular unit cells
Source: Sci Rep. 2020 Feb 14;10:2658. doi: 10.1038/s41598-020-59537-8 (PMC7021700; doi:10.1038/s41598-020-59537-8)
Supplement: Supplementary file 1 — Supplementary Information. [file 41598_2020_59537_MOESM1_ESM.pdf]

[Supplementary Information]

Similarity of therapeutic networks induced by a multi-component herbal remedy, Ukgansan, in neurovascular unit cells

Bu-Yeo Kim[1,*]; Hye-Sun Lim[1]; Yu Jin Kim[1,2]; Eunjin Sohn [1]; Yun Hee Kim[1]; Imhoi Koo[3]; Soo-Jin Jeong[1,*]

[1] Herbal Medicine Research Division, Korea Institute of Oriental Medicine, 1672 Yuseong-daero, Yuseong-gu, Daejeon, 34054, Republic of Korea

[2] College of Pharmacy, Chungnam National University, 99 Daehak-ro, Yuseong-gu, Daejeon 34134, Republic of Korea

[3] Huck Institutes of Life Sciences, Pennsylvania State University, University Park, PA 16802, United States of America

[*] Co-correspondence

Bu-Yeo Kim, PhD: buykim@kiom.re.kr

Soo-Jin Jeong, PhD: sjijeong@kiom.re.kr

Herbal Medicine Research Division, Korea Institute of Oriental Medicine,

1672 Yuseong-daero, Yuseong-gu, Daejeon, 34054, Republic of Korea

Phone: +82 42 868 9401, Fax: + 82 42 868 947

Supplementary Table 1. Biological properties of the herbal components of UGS.

Scientific name	Symbol	Biological properties	Supplementary References
Uncaria sinensis	C1	Neuroprotection, Anti neuroinflammation	[1~4]
Atractylodes japonica	C2	Neuroprotection, Anti neuroinflammation, Anti nociception, Promote differentiation	[5~9]
Poria cocos	C3	Neuroprotection, Immune modulation	[10~12]
Bupleurum falcatum	C4	Anti neuroinflammation, Antioxidant, Antidepressant	[13~17]
Angelica gigas	C5	Neuroprotection, Anti inflammation, Growth inhibition	[18~21]
Cnidium officinale	C6	Antioxidant, Anti neuroinflammation, Anticancer	[22~26]
Glycyrrhiza uralensis	C7	Bioactive Constituents, Anti inflammation, Detoxification, Immune modulation	[27~30]

Supplementary References

[1] Seo, H. B. et al. Partially purified components of Uncaria sinensis attenuate blood brain barrier disruption after ischemic brain injury in mice. *BMC Complement. Altern. Med.* **15,** 157 (2015).

[2] Kang, B. K. et al. Anti-Neuroinflammatory Effects of Uncaria sinensis in LPS-Stimulated BV2 Microglia Cells and Focal Cerebral Ischemic Mice. *Am. J. Chin. Med.* **43,** 1099-115 (2015).

[3] Jang, J. Y. et al. Hexane extract from Uncaria sinensis exhibits anti-apoptotic properties against glutamate-induced neurotoxicity in primary cultured cortical neurons. *Int. J. Mol. Med.* **30,** 1465-1472 (2012).

[4] Shi, Z. et al Neuroprotective effects of aqueous extracts of Uncaria tomentosa: Insights from 6-OHDA induced cell damage and transgenic Caenorhabditis elegans model. *Neurochem. Int.* **62,** 940-947 (2013).

[5] Han, Y., Jung, H. W. & Park, Y. K. The roots of Atractylodes japonica Koidzumi promote adipogenic differentiation via activation of the insulin signaling pathway in 3T3-L1 cells. *BMC Complement. Altern. Med.* **12,** 154 (2012).

[6] Chen, L. G. et al. Anti-inflammatory and Antinociceptive Constituents of Atractylodes japonica Koidzumi. *J. Agric. Food. Chem.* **64,** 2254-2262 (2016).

[7] Liu, C., Zhao, H., Ji, Z. H. & Yu, X. Y. Neuroprotection of atractylenolide III from Atractylodis macrocephalae against glutamate-induced neuronal apoptosis via inhibiting caspase signaling pathway. *Neurochem. Res.* **39,** 1753-1758 (2014).

[8] Gao, Q., Ji, Z. H., Yang, Y., Cheng, R. & Yu, X. Y. Neuroprotective effect of Rhizoma Atractylodis macrocephalae against excitotoxicity-induced apoptosis in cultured cerebral cortical neurons. *Phytother. Res.* **26,** 557-561 (2012).

[9] Zhao, H., Ji, Z. H., Liu, C. & Yu, X. Y. Neuroprotection and mechanisms of atractylenolide III in preventing learning and memory impairment induced by chronic high-dose homocysteine administration in rats. *Neuroscience* **290,** 485-491 (2015).

[10] Park, Y. H. et al. Poria cocos water extract (PCW) protects PC12 neuronal cells from beta-amyloid-induced cell death through antioxidant and antiapoptotic functions. *Pharmazie.* **64,** 760-764 (2009).

[11] Ríos, J. L. Chemical constituents and pharmacological properties of Poria cocos. *Planta Med.* **77,** 681-691 (2011).

[12] Cheng, S., Eliaz, I., Lin, J., Thyagarajan-Sahu, A. & Sliva, D. Triterpenes from Poria cocos suppress growth and invasiveness of pancreatic cancer cells through the downregulation of MMP-7. *Int. J. Oncol.* **42,** 1869-1874 (2013).

[13] Lee, B., Yun, H. Y., Shim, I., Lee, H. & Hahm, D. H. Bupleurum falcatum prevents depression and anxiety-like behaviors in rats exposed to repeated restraint stress. *J. Microbiol. Biotechnol.* **22,** 422-430 (2012).

[14] Kim, S. M., Kim, S. C., Chung, I. K., Cheon, W. H. & Ku, S. K. Antioxidant and Protective Effects of Bupleurum falcatum on the L-Thyroxine-Induced Hyperthyroidism in Rats. *Evid. Based Complement. Alternat. Med.* **2012,** 578497 (2012).

[15] Park, W. H. et al. Ethanol extract of Bupleurum falcatum and saikosaponins inhibit neuroinflammation via inhibition of NF-κB. *J. Ethnopharmacol.* **174,** 37-44 (2015).

[16] Kwon, S. et al. Antidepressant-like effect of the methanolic extract from Bupleurum falcatum in the tail suspension test. *Prog. Neuropsychopharmacol. Biol. Psychiatry* **34,** 265-270 (2010).

[17] Lee, J. Y., Kim, H. S., Oh, T. H. & Yune, T. Y. Ethanol Extract of Bupleurum falcatum Improves Functional Recovery by Inhibiting Matrix Metalloproteinases-2 and -9 Activation and Inflammation after Spinal Cord Injury. *Exp. Neurobiol.* **19,** 146-154 (2010).

[18] Cho, J. H., Kwon, J. E., Cho, Y., Kim, I. & Kang, S. C. Anti-Inflammatory Effect of Angelica gigas via Heme Oxygenase (HO)-1 Expression. *Nutrients* **7,** 4862-4874 (2015).

[19] Sowndhararajan, K. & Kim, S. Neuroprotective and Cognitive Enhancement Potentials of Angelica gigas Nakai Root: A Review. *Sci. Pharm.* **85,** pii: E21 (2017).

[20] Jeon, Y. J., et al. Angelica gigas Nakai Has Synergetic Effects on Doxorubicin-Induced Apoptosis. *Biomed. Res. Int.* **2018,** 6716547 (2018).

[21] Jang, J. Y. et al. An ethanolic extract of Angelica gigas improves atherosclerosis by inhibiting vascular smooth muscle cell proliferation. *Lab. Anim. Res.* **30,** 84-89 (2014).

[22] Ramalingam, M. & Yong-Ki, P. Free radical scavenging activities of Cnidium officinale Makino and Ligusticum chuanxiong Hort. methanolic extracts. *Pharmacogn. Mag.* **6,** 323-330 (2010).

[23] de la Cruz, J., Kim, D. H. & Hwang, S. G. Anticancer effects of Cnidium officinale Makino extract mediated through apoptosis and cell cycle arrest in the HT-29 human colorectal cancer cell line. *Asian Pac. J. Cancer Prev.* **15,** 5117-5121 (2014).

[24] Lee, Y. M. et al. Cnidium officinale extract and butylidenephthalide inhibits retinal neovascularization in vitro and in vivo. *BMC Complement. Altern. Med.* **16,** 231 (2016).

[25] Bae, K. E., Choi, Y. W., Kim, S. T. & Kim, Y. K. Components of rhizome extract of Cnidium officinale Makino and their in vitro biological effects. *Molecules* **16,** 8833-8847 (2011).

[26] Lee, S. H. et al. Ethanol extract of Cnidium officinale exhibits anti-inflammatory effects in BV2 microglial cells by suppressing NF-κB nuclear translocation and the activation of the PI3K/Akt signaling pathway**.** *Int. J. Mol. Med.* **32,** 876-882 (2013).

[27] Tanemoto, R. et al. The constituents of licorice (Glycyrrhiza uralensis) differentially suppress nitric oxide production in interleukin-1β-treated hepatocytes. *Biochem. Biophys. Rep.* **2,** 153-159 (2015).

[28] Ji, S. et al. Bioactive Constituents of Glycyrrhiza uralensis (Licorice): Discovery of the Effective Components of a Traditional Herbal Medicine. *Nat. Prod.* **79,** 281-292 (2016).

[29] Yang, N. et al. Glycyrrhiza uralensis flavonoids present in anti-asthma formula, ASHMI™, inhibit memory Th2 responses in vitro and in vivo. *Phytother. Res.* **27,** 1381-1391 (2013).

[30] Gong, H. et al. A protective mechanism of licorice (Glycyrrhiza uralensis): isoliquiritigenin stimulates detoxification system via Nrf2 activation. *J. Ethnopharmacol.* **162,** 134-139 (2015).

Supplementary Table 2. List of enriched pathways from SPIA based on the phosphorylation levels of signaling proteins.

	FDR		
**Pathways**	**HT22**	**BV-2**	**HBVP**
Pathways in cancer	7.13E-14	6.81E-17	2.09E-04
Viral carcinogenesis	1.44E-09	2.23E-07	7.80E-03
Pancreatic cancer	7.15E-09	1.20E-06	1.07E-03
Prostate cancer	1.60E-08	2.11E-10	4.62E-04
Focal adhesion	1.90E-07	2.74E-07	1.77E-02
ErbB signaling pathway	3.06E-07	9.01E-10	2.52E-02
Osteoclast differentiation	3.06E-07	1.92E-07	3.02E-02
Acute myeloid leukemia	4.68E-07	1.92E-07	4.81E-03
Chemokine signaling pathway	4.68E-07	2.74E-07	2.52E-02
Epstein-Barr virus infection	4.68E-07	6.06E-11	5.52E-03
Tuberculosis	4.68E-07	3.84E-07	1.52E-02
MAPK signaling pathway	6.24E-07	6.91E-10	4.81E-03
Endometrial cancer	4.32E-06	1.79E-04	1.05E-02
Chronic myeloid leukemia	4.32E-06	6.00E-06	3.00E-04
Neurotrophin signaling pathway	7.91E-06	1.01E-06	1.62E-03
HTLV-I infection	2.07E-05	3.65E-06	6.83E-04
Measles	2.26E-05	2.97E-06	4.17E-02
VEGF signaling pathway	2.76E-05	1.01E-06	8.81E-03
Cytokine-cytokine receptor interaction	4.74E-05	9.36E-03	1.49E-02
Adipocytokine signaling pathway	5.09E-05	3.50E-03	4.62E-04
Hepatitis C	5.17E-05	6.92E-07	5.52E-03
Epithelial cell signaling in Helicobacter pylori infection	6.83E-05	9.33E-05	2.23E-02
Influenza A	9.81E-05	1.32E-05	8.81E-03
Toxoplasmosis	1.38E-04	1.92E-07	5.46E-03
Small cell lung cancer	1.61E-04	4.70E-03	1.53E-03
Glioma	1.95E-04	1.86E-04	2.19E-02
Insulin signaling pathway	1.86E-04	4.28E-07	1.07E-03
Regulation of actin cytoskeleton	1.91E-04	3.25E-03	2.44E-02
Non-small cell lung cancer	2.01E-04	2.16E-03	2.87E-02
Colorectal cancer	2.24E-04	5.26E-04	2.39E-03
Cell cycle	2.58E-04	8.97E-05	5.52E-03
Jak-STAT signaling pathway	2.46E-04	2.27E-03	
GnRH signaling pathway	2.67E-04	5.60E-06	
Chagas disease (American trypanosomiasis)	6.35E-04	2.07E-03	4.81E-03
Transcriptional misregulation in cancer	6.35E-04	2.67E-02	2.19E-02
Herpes simplex infection	6.60E-04	7.00E-03	2.35E-02
T cell receptor signaling pathway	8.84E-04	4.57E-07	2.87E-02
Amyotrophic lateral sclerosis (ALS)	1.04E-03	9.39E-04	1.38E-02
Calcium signaling pathway	1.56E-03	2.69E-02	2.37E-02
Natural killer cell mediated cytotoxicity	1.30E-03	2.07E-04	
Shigellosis	1.76E-03	5.54E-03	4.56E-03
Pathogenic Escherichia coli infection	1.78E-03		5.52E-03
mTOR signaling pathway	1.92E-03	1.29E-02	1.62E-03
Tight junction	2.28E-03	3.49E-02	2.56E-03
B cell receptor signaling pathway	3.35E-03	2.54E-04	1.77E-02
Toll-like receptor signaling pathway	3.55E-03	1.54E-04	4.95E-03

	FDR		
**Pathways**	**HT22**	**BV-2**	**HBVP**
Melanoma	3.85E-03	3.45E-04	2.47E-02
Leukocyte transendothelial migration	5.51E-03		1.85E-02
Apoptosis	5.54E-03	3.01E-02	4.64E-03
Bacterial invasion of epithelial cells	5.54E-03	2.89E-02	
NF-kappa B signaling pathway	6.32E-03	2.16E-05	2.52E-02
Aldosterone-regulated sodium reabsorption	5.08E-03	4.79E-02	5.52E-03
Oocyte meiosis	8.07E-03	1.34E-03	2.52E-02
Dopaminergic synapse	8.78E-03	7.65E-03	1.31E-02
Wnt signaling pathway	9.25E-03	3.61E-02	1.44E-02
Amphetamine addiction	1.31E-02		9.60E-03
Fc epsilon RI signaling pathway	1.31E-02	1.30E-05	4.38E-02
Fc gamma R-mediated phagocytosis	1.59E-02	1.34E-03	4.13E-02
Progesterone-mediated oocyte maturation	1.59E-02	2.16E-05	1.22E-02
Bladder cancer	1.62E-02	1.38E-02	
Melanogenesis	1.83E-02	8.28E-03	4.38E-02
Cocaine addiction	1.93E-02	3.85E-02	1.77E-02
Alzheimer's disease	1.94E-02	3.10E-02	9.60E-03
Gap junction	1.74E-02	3.49E-02	1.67E-02
Amoebiasis	1.76E-02		
Leishmaniasis	1.76E-02	6.96E-03	
Gastric acid secretion	1.43E-02		
Huntington's disease	1.67E-02		5.46E-03
Pertussis	1.87E-02	1.86E-02	
Viral myocarditis	1.87E-02		9.60E-03
Renal cell carcinoma	2.37E-02	1.29E-02	
Thyroid cancer	2.55E-02		2.19E-02
Salmonella infection	2.83E-02	3.41E-03	
Cholinergic synapse	2.89E-02		
TGF-beta signaling pathway	2.06E-02		2.42E-02
Long-term depression	2.19E-02	1.34E-03	3.14E-02
NOD-like receptor signaling pathway	2.66E-02	5.37E-04	2.23E-02
Long-term potentiation	3.86E-02	8.31E-03	2.63E-02
p53 signaling pathway	4.46E-02	1.41E-02	1.76E-02
Cytosolic DNA-sensing pathway	4.57E-02	1.25E-02	5.52E-03
Alcoholism	4.41E-02	3.94E-05	1.49E-02
Vascular smooth muscle contraction		1.03E-03	3.96E-02
Type II diabetes mellitus		2.23E-02	2.39E-03
Axon guidance		1.34E-02	
Antigen processing and presentation		2.56E-02	
RIG-I-like receptor signaling pathway		2.74E-02	1.38E-02
Retrograde endocannabinoid signaling		2.39E-02	
Glutamatergic synapse		2.52E-02	
Dorso-ventral axis formation		3.54E-02	
Arrhythmogenic right ventricular cardiomyopathy (ARVC)		3.08E-02	1.49E-02
Legionellosis			4.81E-03
Parkinson's disease			5.52E-03
Carbohydrate digestion and absorption			2.96E-02

Supplementary Table 3. List of individual chemical compounds included in each functional subcategory of kinase inhibitors.

				Cell cycle inhibitors				Inhibitors of growth factor receptors				MAPK inhibitors			
				CDK inhibitors	ATM kinase inhibitors	Mitotic inhibitors	Other cell cycle inhibitors	EGFR inhibitors	VEGFR inhibitors	FGFR inhibitors	Other inhibitors of growth factor receptors	p38 MAPK inhibitors	MEK inhibitors	RAF inhibitors	Other MAPK inhibitors
Cell cycle inhibitors	CDK inhibitors	K07762753	aminopurvalanol-a	O											
		K31268420	NSC-693868	O											
		K37312348	kenpaullone	O											
		K51276371	aloisine	O											
		K51313569	palbociclib	O											
		K64800655	PHA-793887	O											
		K71726959	BRD-K71726959	O											
		K72726508	arcyriaflavin-a	O											
		K72783841	tyrphostin-AG-555	O											
		K87932577	CDK1-5-inhibitor	O											
	ATM kinase inhibitors	A11678676	wortmannin		O										
		A50737080	CGK-733		O										
		K02404261	caffeine		O										
		K15592317	CP466722		O										
		K25311561	KU-55933		O										
	Mitotic inhibitors	A06352508	SB-218078			O									
		K19136521	indirubin			O									
		K46056750	AZD-7762			O									
		K63923597	barasertib			O									
		K83963101	MLN-8054			O									
	Other cell cycle inhibitors	K13514097	everolimus				O								
		K13646352	midostaurin				O								
		K29733039	deforolimus				O								
		K35687265	ON-01910				O								
		K84937637	sirolimus				O								
Inhibitors of growth factor receptors	EGFR inhibitors	K03670461	tyrphostin-AG-82					O							
		K08132273	tyrphostin					O							
		K11158509	tyrphostin-B44					O							
		K13087974	4,5-dianilinophthalimide					O							
		K14441456	tyrphostin-AG-556					O							
		K16977723	PP-3					O							
		K19687926	lapatinib					O							
		K21853356	RG-14620					O							
		K23583188	lavendustin-a					O							
		K32292990	CGP-53353					O							
		K32906660	bis-tyrphostin					O							
		K34533029	tyrphostin-AG-494					O							
		K43797669	genistein					O							
		K47943470	tyrphostin-51					O							
		K49657628	tyrphostin-AG-18					O							
		K50168500	canertinib					O							
		K59469039	AG-879					O							
		K64052750	gefitinib					O							
		K66175015	afatinib					O							
		K68336408	tyrphostin-AG-1478					O							
		K68407802	KIN001-055					O							
		K70401845	erlotinib					O							
		K70914287	BIBX-1382					O							
		K72420232	WZ-4002					O							
		K73293050	WZ-3146					O							
		K76908866	CP-724714					O							
		K79930101	GW-583340					O							
		K80725632	lavendustin-c					O							
		K81209512	AG-494					O							
		K82688027	RG-13022					O							
		K85606544	neratinib					O							
		K88741031	methyl-2,5-dihydroxycinnamate					O							
		K96778649	tyrphostin-47					O							
		U08759356	EI-346-erlotinib-analog					O							
		U25771771	WZ-4-145					O							
	VEGFR inhibitors	K20287671	SU-4312						O						
		K34995470	SU-1498						O						
		K41337261	ZM-306416						O						
		K51816706	oxindole-I						O						
		K63504947	semaxanib						O						
		K67831364	ZM-323881						O						
		K96084870	DMBI						O						
	FGFR inhibitors	K07881437	danusertib							O					
		K71035033	masitinib							O					
		K85402309	dovitinib							O					
		K91696562	orantinib							O					
		K96862998	pirfenidone							O					
		K97764662	PD-173074							O					
	Other inhibitors of growth factor receptors	K02965346	SU-11274								O				
		K04146668	GW-441756								O				
		K07888196	tyrphostin-AG-538								O				
		K23192422	lestaurtinib								O				
		K78431006	crizotinib								O				
MAPK inhibitors	p38 MAPK inhibitors	A37704979	SB-203580									O			
		K05464208	JX-401									O			
		K06234293	LY-364947									O			
		K07736136	VX-702									O			
		K42452249	EO-1428									O			
		K52751261	TAK-715									O			
		K54330070	SB-202190									O			
		K82091397	SB-239063									O			
		K85871428	SC-68376									O			
		K91900765	VX-745									O			
		K95202259	ML-3163									O			
		K96809896	SKF-86002									O			
	MEK inhibitors	K05104363	PD-184352										O		
		K12244279	MEK1-2-inhibitor										O		
		K18787491	U-0126										O		
		K49865102	PD-0325901										O		
		K88677950	PD-198306										O		
		K89014967	AS-703026										O		
	RAF inhibitors	K01253243	SB-590885											O	
		K05804044	AZ-628											O	
		K07859598	GW-5074											O	
		K16478699	PLX-4720											O	
		K23984367	sorafenib											O	
		K74514084	pazopanib											O	
	Other MAPK inhibitors	K31283835	tofacitinib												O
		K42500029	CGP-57380												O
		K50387473	XMD-892												O
		K64857848	XMD-885												O
		K68756823	FR-180204												O

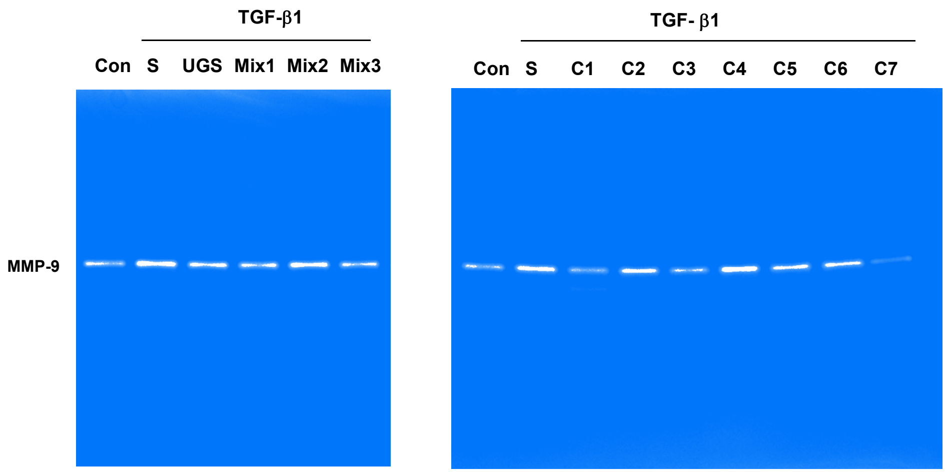

Supplementary Fig. S1. Full length images of gels of Fig. 1c. HBVP cells were treated with TGF-β1 (10 ng/mL) and each herbal extract for 24 h. MMP-9 enzymatic activity was assessed by gelatin zymography using the culture supernatants of each sample. Areas of gelatinase activity were detected as clear bands against the blue-stained gelatin background. Con represents untreated control samples and S represents TGF-β1-treated samples.

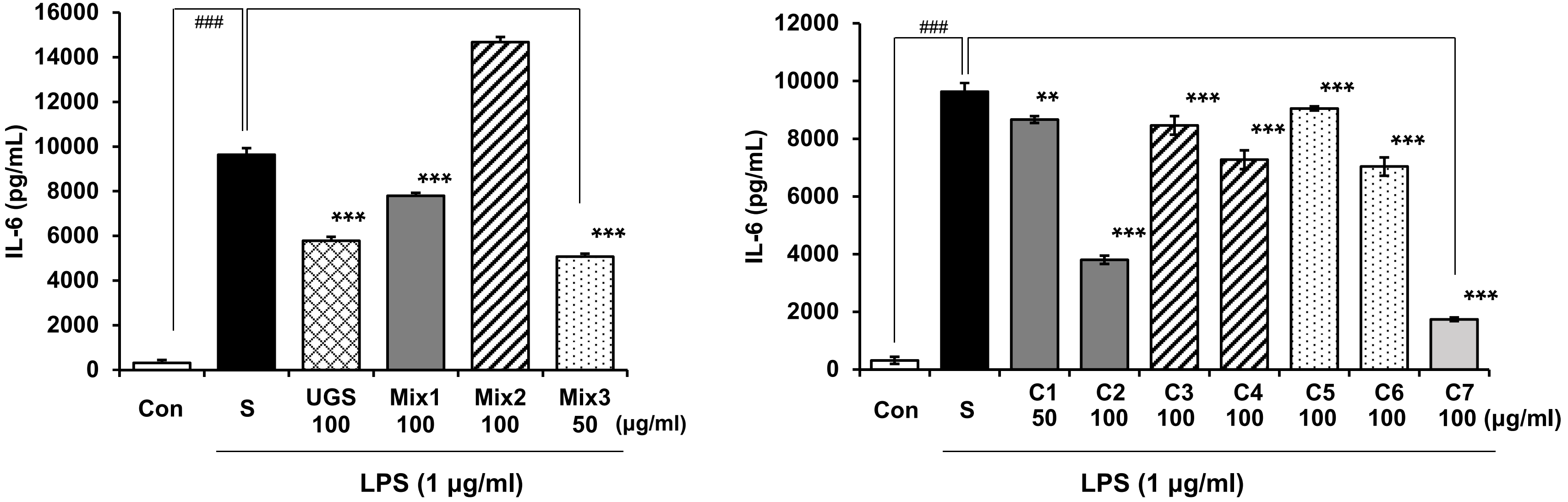

Supplementary Fig. S2. Effects of UGS and its herbal components on LPS-stimulated BV-2 microglia. BV-2 microglial cells were pretreated with each herbal extract for 2 h and then stimulated with LPS (1 μg/mL) for an additional 22 h. The supernatants were collected and applied to ELISA for IL-6. The left panel presents the effects of UGS and the 3 herbal component mixtures (Mix), while the right panel presents the effects of individual herbal components (C1–C7). The white bar (Con) represents untreated control samples and the black bar (S) represents LPS-stimulated samples. Data represent the mean ± standard error of 3 independent experiments. ###p < 0.01 vs untreated cells; *p < 0.05, **p < 0.01 or ***p < 0.001 vs LPS-stimulated cells.

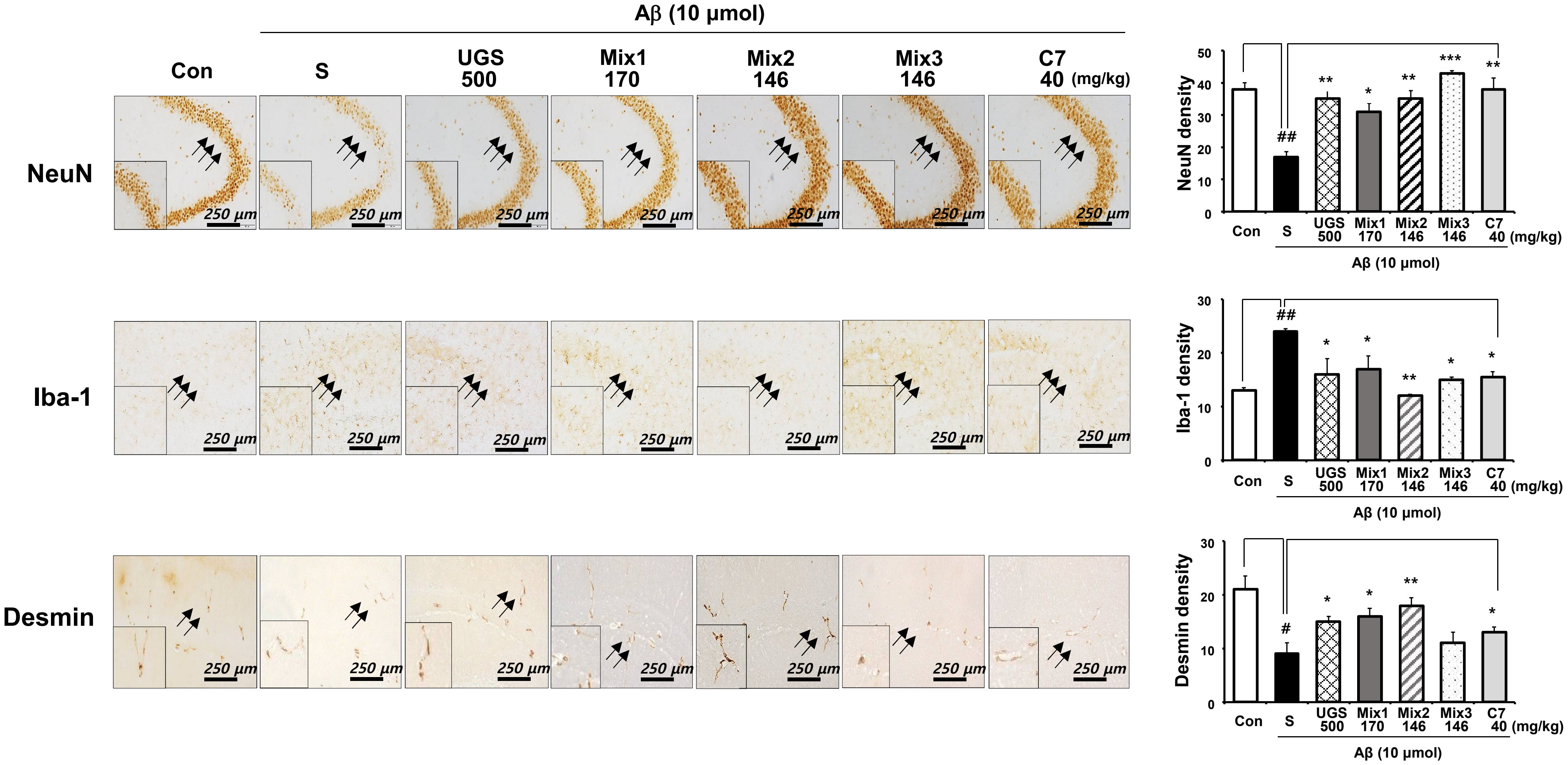

Supplementary Fig. S3. Effects of UGS and its herbal components on the Aβ-induced mouse model of Alzheimer's disease. For the immunohistochemistry (IHC) assay, Aβ aggregates (10 μmol per 10% dimethyl sulfoxide in PBS) were injected into the intracerebroventricular (ICV) region of ICR mice in stereotaxic coordinates. Vehicle or each herbal extract was orally administered for 21 days. Expressions of NeuN and desmin in the hippocampus and Iba-1 in the cortex were determined by IHC (magnification, ×200) and then quantified using ImageJ as shown in right panel. Data represent the mean ± standard error of 3 independent experiments. ###p < 0.01 vs untreated samples; *p < 0.05, **p < 0.01 or ***p < 0.001 vs Aβ-stimulated samples. The length of the scale bar indicates 250 μm.

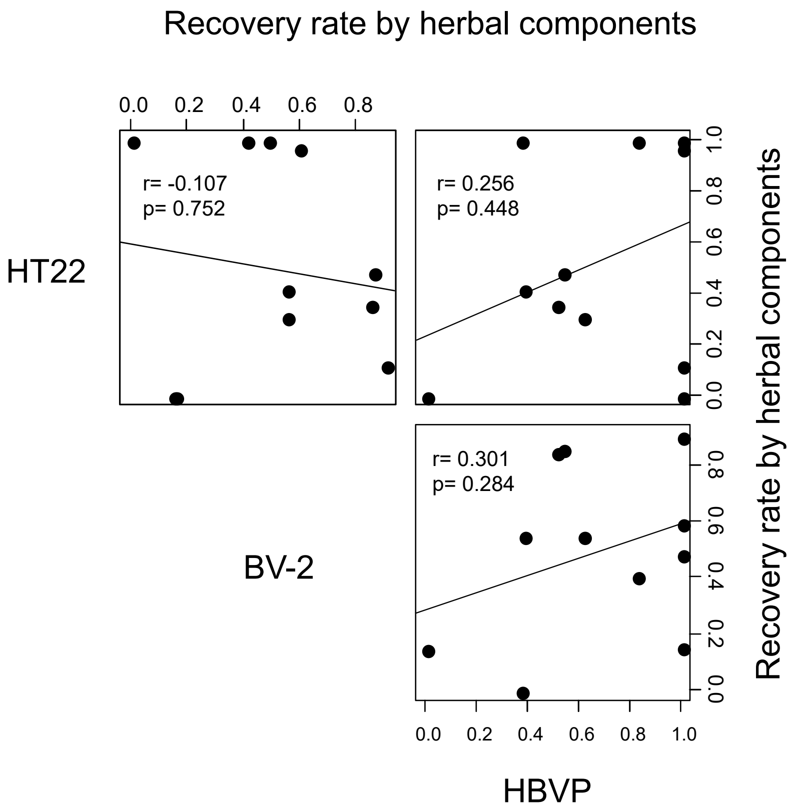

Supplementary Fig. S4. Therapeutic similarities between neurovascular cell types based on in vitro experiments. HT22, BV-2, and HBVP cells exposed to H_2O_2, LPS, and TGF-β1, respectively were treated with each herbal component. For HT22 cells, cytotoxicity was determined by the CCK assay. For BV-2 cells, the amount of TNF-α was measured. For HBVP cells, MMP-9 enzymatic activity was assessed. The therapeutic effect of each herbal component was measured as recovery ratio compared with untreated cells, then compared among neurovascular cell types.

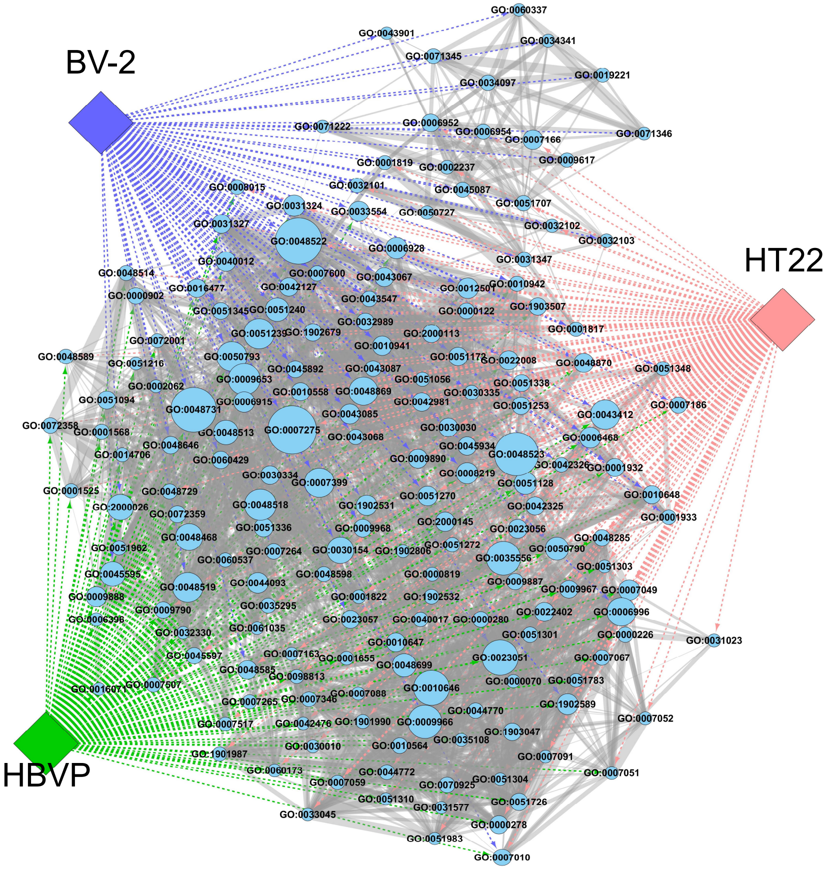

Supplementary Fig. S5. Functional connections between neurovascular cells based on gene expression. HT22, BV-2, and HBVP cells were exposed to H2O2, LPS, and TGF-β1, respectively. Total RNA extracts from cultivated cells were analyzed by RNA-seq technology. Gene expression levels were compared with those of control cells to obtain expression ratios. Genes showing expression ratios above 2 or below 0.5 in each experimental condition were identified as DEGs. GO terms associated with DEGs from each stimulated neurovascular cell type were identified with DAVID, and redundancies removed using ReviGO. The resultant GO terms were connected with each other based on the common genes ($p < 0.001$) included in each pair of GO terms, as determined by the Enrichment Map. Node size and edge thickness represent the number of genes included in a GO term and shared by a pair of GO terms, respectively.

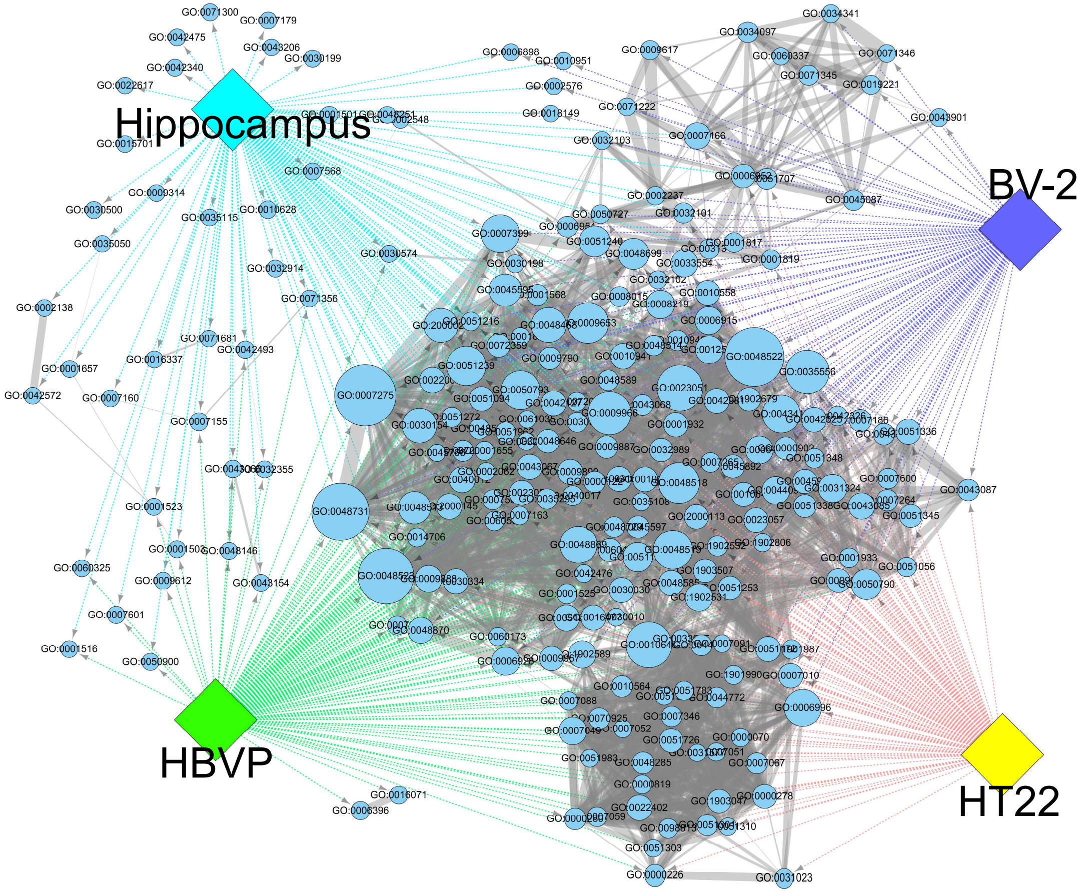

Supplementary Fig. S6. Functional connections between neurovascular cells and mouse hippocampus based on gene expression. HT22, BV-2, and HBVP cells were exposed to H_2O_2, LPS, and TGF-β1, respectively. Mice were injected with Aβ into the intracerebroventricular (ICV) region. Total RNA extracts from cultivated cells and mouse hippocampus were analyzed by RNA-seq technology. Gene expression levels were compared with those of control cells to obtain expression ratios. Genes showing expression ratios above 2 or below 0.5 in each experimental condition were identified as DEGs. GO terms associated with DEGs from each stimulated neurovascular cell type were identified with DAVID and redundancies removed using ReviGO. The resultant GO terms were connected with each other based on the common genes ($p < 0.001$) included in each pair of GO terms, as determined by the Enrichment Map. Node size and edge thickness represent the number of genes included in a GO term and shared by a pair of GO terms, respectively.

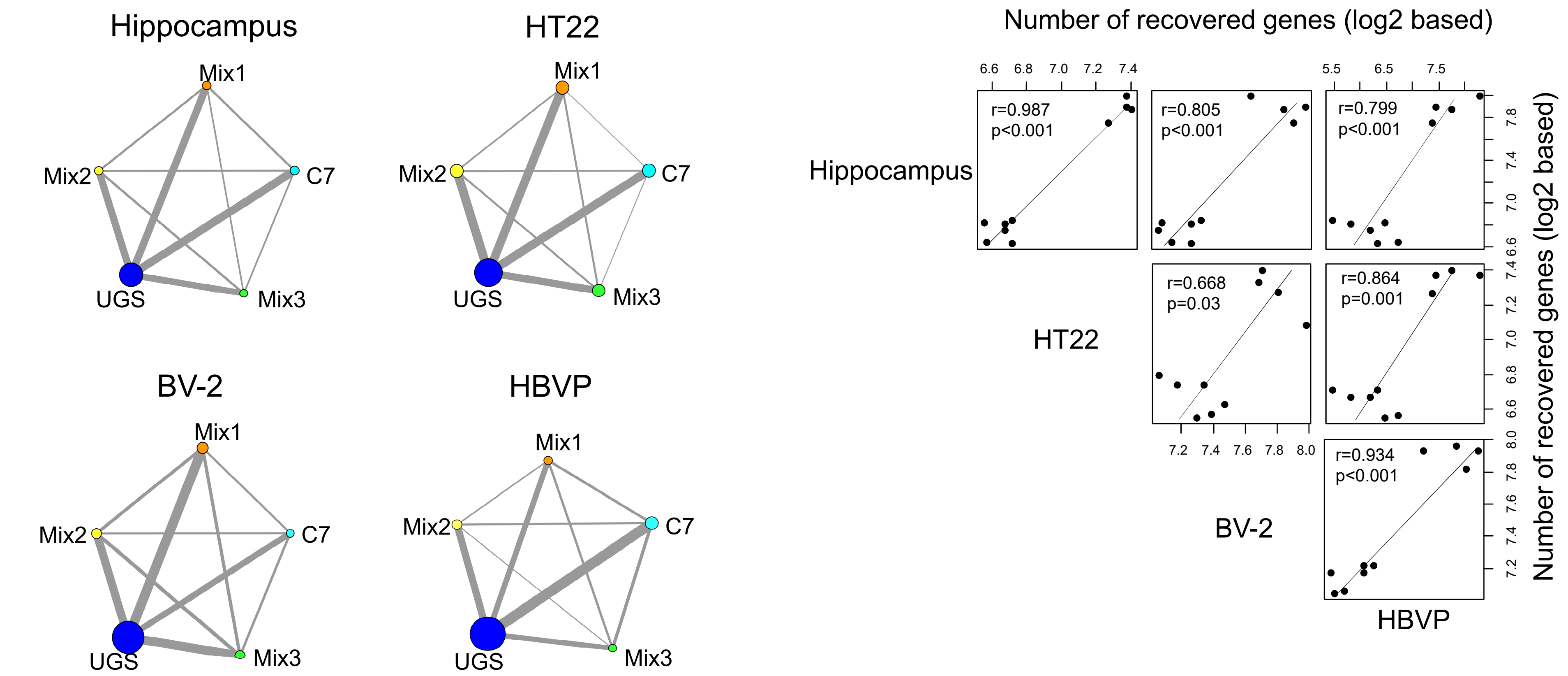

Supplementary Fig. S7. Therapeutic similarities between neurovascular cell types based on gene expression. Gene expression ratios were measured in HT22, BV-2, and HBVP cells exposed to H_2O_2, LPS, and TGF-β1, respectively, by RNA-seq technology. For hippocampal tissue, mice were injected with Aβ into the intracerebroventricular (ICV) region. DEGs were defined as genes showing expression ratios above 2 or below 0.5 in each stimulated neurovascular cell type. Recovered genes were defined as those whose expression levels were restored to normal level (expression ratio between 0.5 and 2). By measuring the similarity between each pair of herbal components (Mix1, Mix2, Mix3, and C7) in terms of commonly recovered genes, therapeutic networks were constructed. Node size and edge thickness represent the number of genes recovered by each herbal component and the number of shared recovered genes between two herbal components, respectively. The structure of the therapeutic network obtained from each neurovascular cell type was then compared with those from other cell types to obtain correlation patterns between neurovascular cell types.

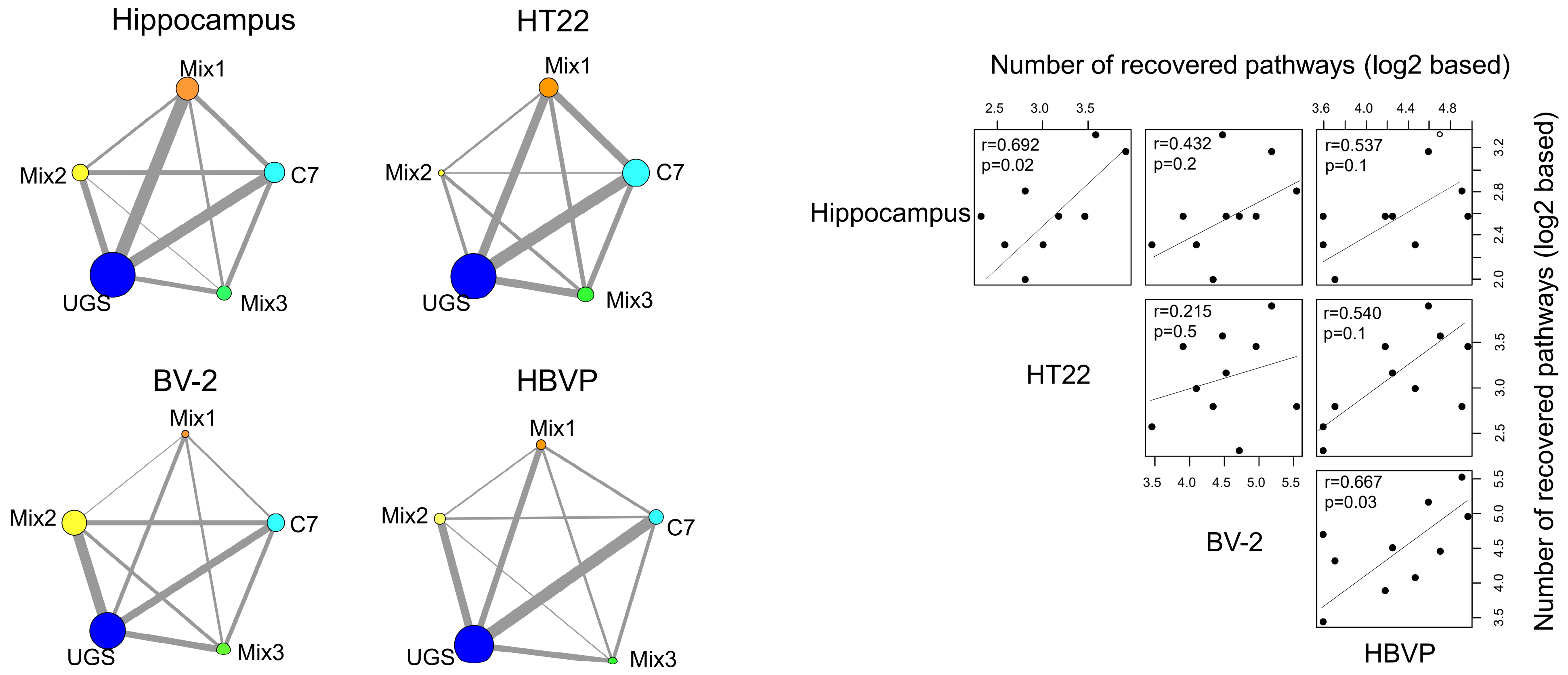

Supplementary Fig. S8. Therapeutic similarities between neurovascular cells based on pathway activity. Pathway activities from stimulated neurovascular cells were calculated by linearly combining the expression levels of the genes included in each pathway. As in the case of gene expression, by measuring the similarity between each pair of herbal components in terms of commonly recovered pathways, therapeutic networks were constructed. Differentially regulated pathways were selected as pathways with FDR ≤ 0.01 in stimulated neurovascular cells. Among such differentially regulated pathways, a 50% reduction in activity by herbal components was considered pathway activity recovery. Node size and edge thickness represent the number of pathways recovered by each herbal component, and the number of shared recovered pathways between two herbal components, respectively. The structure of the therapeutic network obtained from each neurovascular cell type was then compared with those from other cell types to obtain correlation patterns between neurovascular cell types.

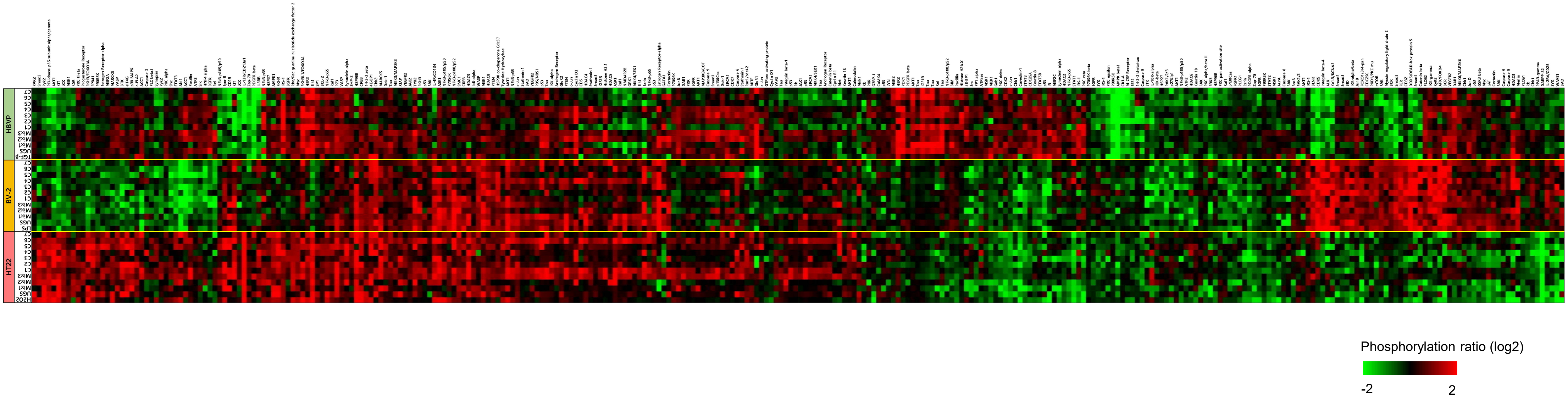

Supplementary Fig. S9. Regulation of phosphorylation by UGS and its components. The phosphorylation status of signaling proteins was measured using antibody array technology in stimulated neurovascular cells. Protein phosphorylation levels were compared with those of control samples to obtain phosphorylation ratios. A phosphorylation profile was obtained by hierarchically clustering approximately 310 proteins showing significant variation (standard deviation > 1.0) over all neurovascular cell types. The red and green colors reflect high and low protein phosphorylation, respectively, as indicated by the scale bar.

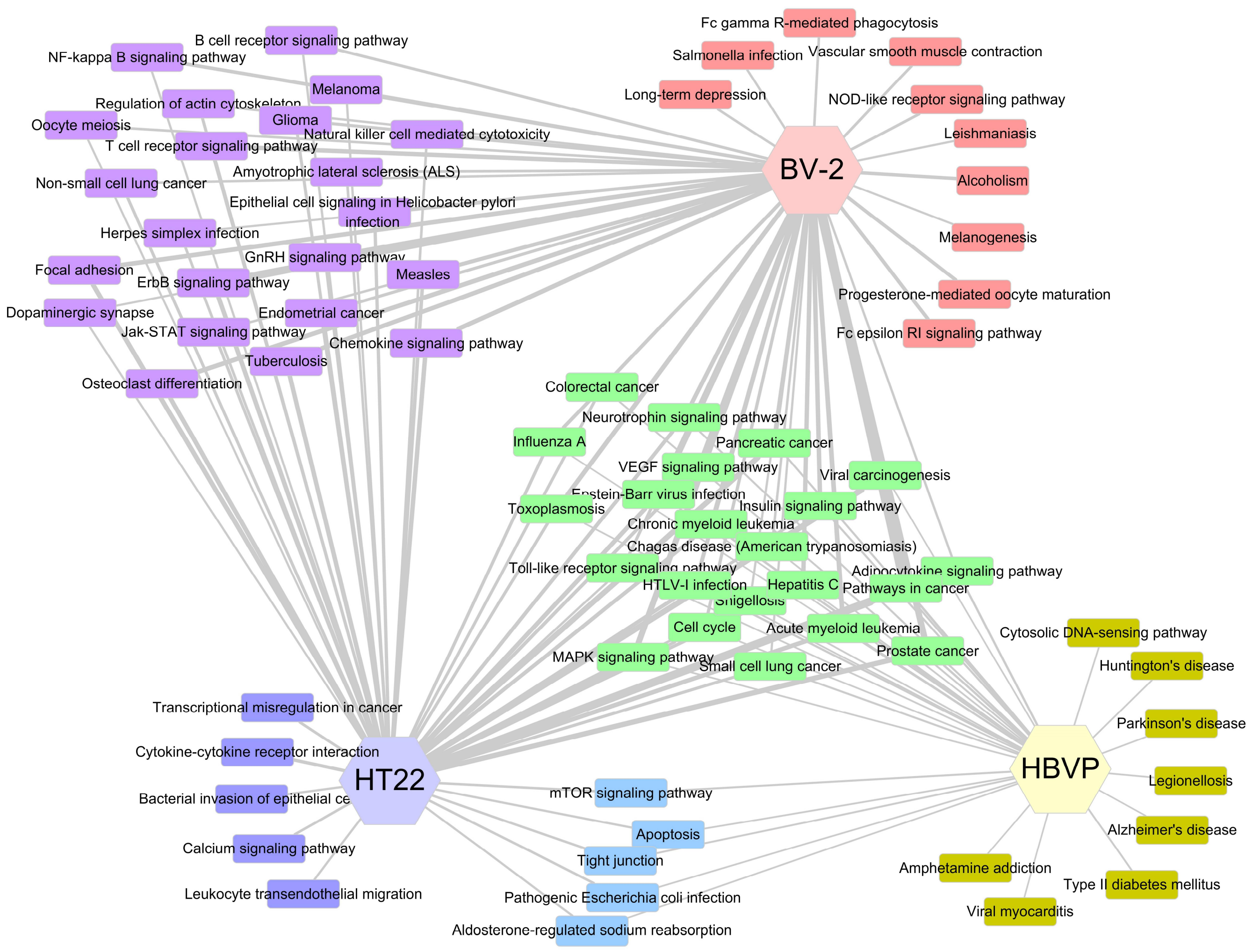

Supplementary Fig. S10. Regulation of phosphorylation by UGS and its components. The distribution of significant pathways (FDR < 0.01) from SPIA was displayed in connection with neurovascular cell types.

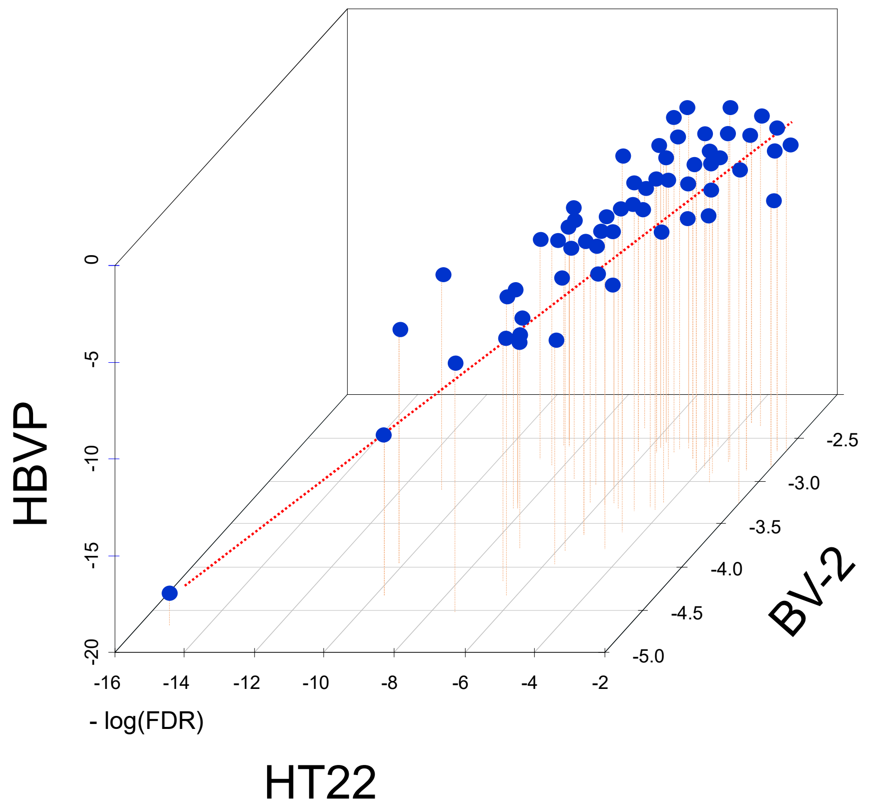

Supplementary Fig. S11. Regulation of phosphorylation by UGS and its components. Relationships between neurovascular cell types were measured in terms of their FDR values for common pathways (FDR < 0.01).

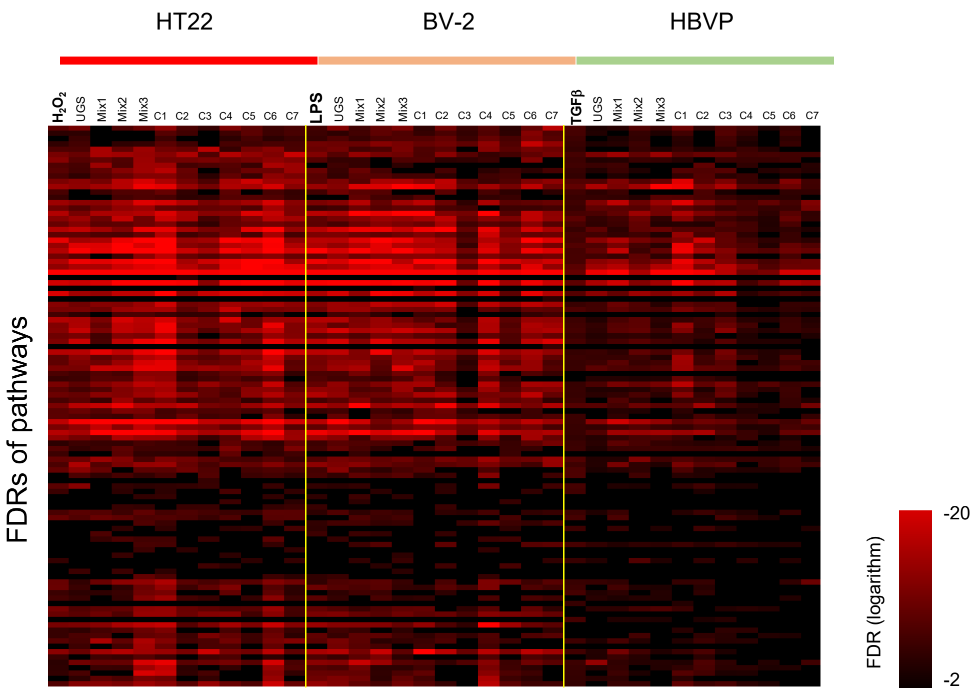

Supplementary Fig. S12. Regulation of phosphorylation by UGS and its components. Enriched phospho-based pathways (FDR < 0.01) from SPIA were hierarchically clustered based on FDR values.

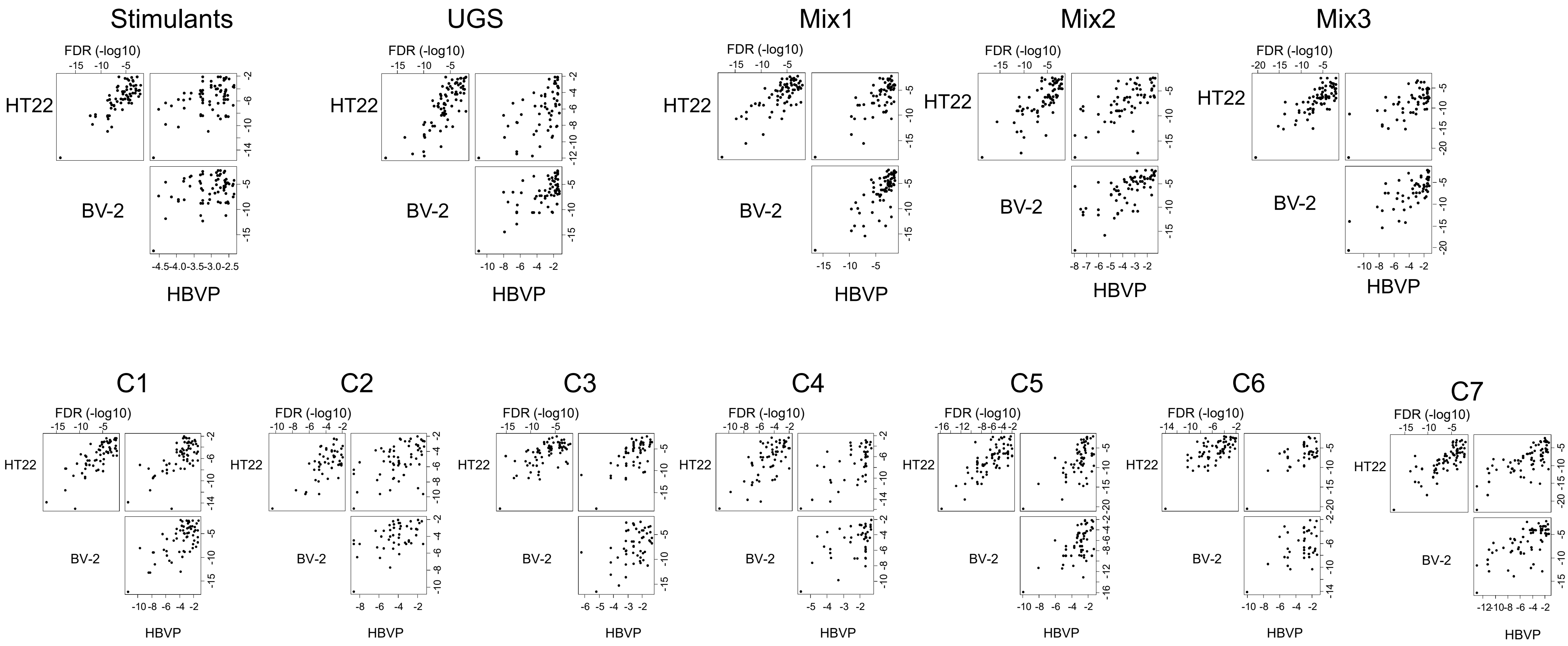

Supplementary Fig. S13. Correlation patterns between neurovascular cell types based on FDR distribution. Enriched phospho-based pathways (FDR < 0.01) in stimulated neurovascular cell types were identified with SPIA. On these enriched phospho-pathways, the effects of herbal components were measured in terms of FDR values. Correlation patterns among FDR values were then measured between neurovascular cell types.

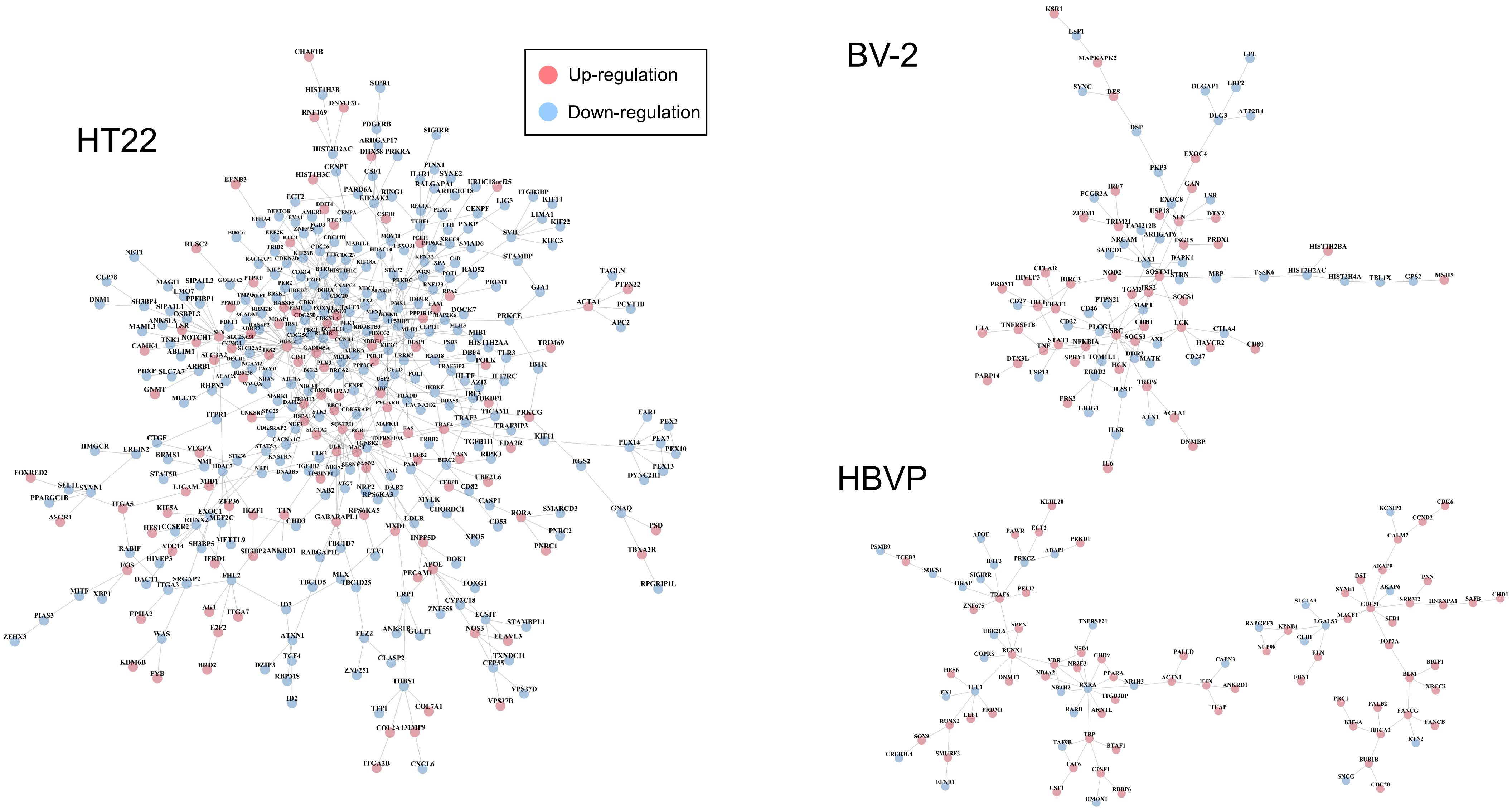

Supplementary Fig. S14. Major modules from neurovascular cells. Modules were identified with the Reactome FI Cytoscape plugin program, using the DEGs from each neurovascular cell type. Expression levels are depicted in red and blue for up- and downregulated genes, respectively.

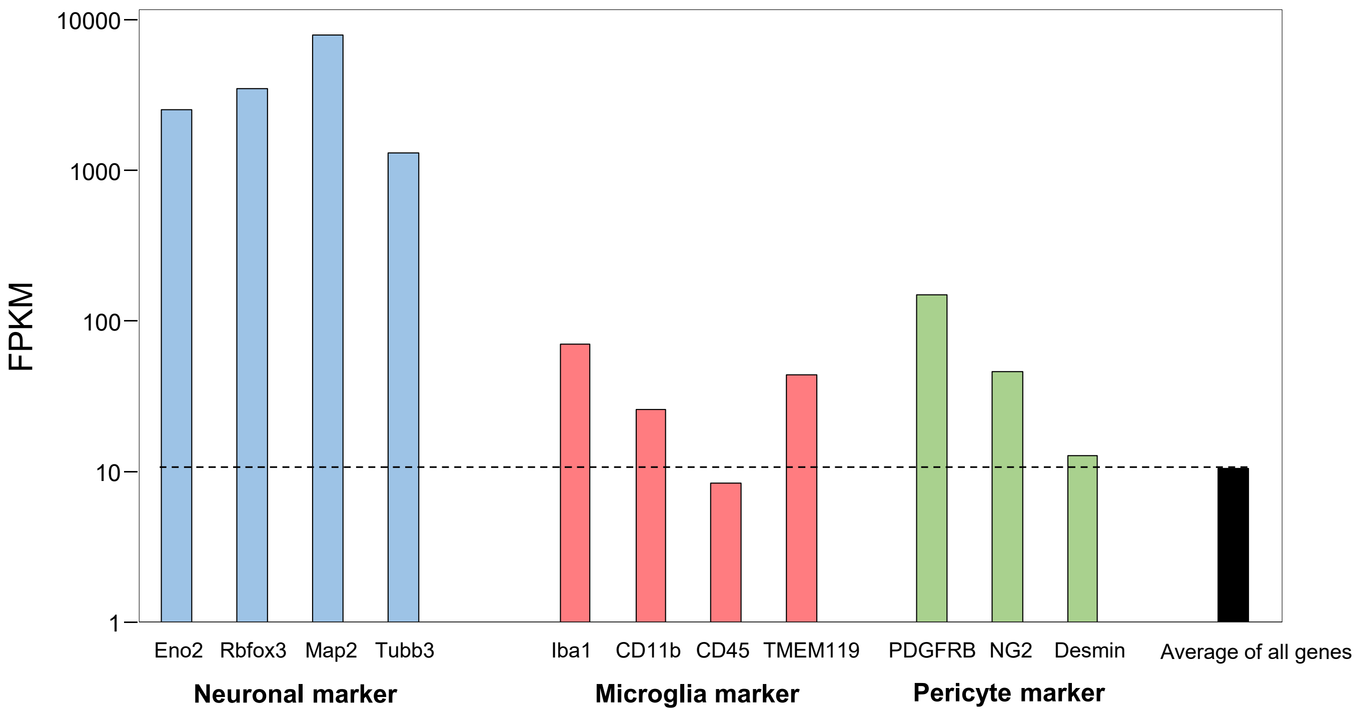

Supplementary Fig. S15. Expressional levels of marker genes of neurovascular cells in hippocampal mouse tissues. Neuronal markers, such as Eno2 (51), Rbfox3 (NeuN) (52), Map2 (53), and Tubb3 (54), were highly expressed when compared with markers of pericytes and Aβ-activated microglia in hippocampal mouse tissues. Gene expression levels were measured by FPKM (Fragments Per Kilobase of transcripts per Million mapped reads) from QuantSeq analysis. Iba1, CD11b, CD45, and TMEM119 were used as markers of activated microglia. PDGFRB, NG2, and desmin were used as markers of pericytes. The averaged value for all spots of genes was used as a control.

51. Sensenbrenner, M., Lucas, M. & Deloulme, J. C. Expression of two neuronal markers, growth-associated protein 43 and neuron-specific enolase, in rat glial cells. J. Mol. Med. 75, 653–663 (1997).
52. Mullen, R. J., Buck, C. R. & Smith, A. M. NeuN, a neuronal specific nuclear protein in vertebrates. Development 116, 201–211 (1992).
53. Izant, J. G. & McIntosh, J. R. Microtubule-associated proteins: a monoclonal antibody to MAP2 binds to differentiated neurons. Proc. Natl. Acad. Sci. U. S. A. 77, 4741–4745 (1980).
54. Lee, M. K., Tuttle, J. B., Rebhun, L. I., Cleveland, D. W. & Frankfurter A. The expression and posttranslational modification of a neuron-specific beta-tubulin isotype during chick embryogenesis. Cell Motil. Cytoskeleton 17, 118–132 (1990).
